# The Natural Stilbenoid (–)-Hopeaphenol Inhibits Cellular Entry of SARS-CoV-2 USA-WA1/2020, B.1.1.7, and B.1.351 Variants

**DOI:** 10.1128/AAC.00772-21

**Published:** 2021-11-17

**Authors:** Ian Tietjen, Joel Cassel, Emery T. Register, Xiang Yang Zhou, Troy E. Messick, Frederick Keeney, Lily D. Lu, Karren D. Beattie, Topul Rali, Pablo Tebas, Hildegund C. J. Ertl, Joseph M. Salvino, Rohan A. Davis, Luis J. Montaner

**Affiliations:** a The Wistar Institute, Philadelphia, Pennsylvania, USA; b Griffith Institute for Drug Discovery, School of Environment and Science, Griffith University, Brisbane, QLD, Australia; c School of Natural and Physical Sciences, The University of Papua New Guinea, Port Moresby, Papua New Guinea; d Perelman School of Medicine, University of Pennsylvania, Philadelphia, Pennsylvania, USA

**Keywords:** COVID-19, SARS-CoV-2, antiviral agents, coronavirus, natural products, stilbenoids

## Abstract

Antivirals are urgently needed to combat the global SARS-CoV-2/COVID-19 pandemic, supplement existing vaccine efforts, and target emerging SARS-CoV-2 variants of concern. Small molecules that interfere with binding of the viral spike receptor binding domain (RBD) to the host angiotensin-converting enzyme II (ACE2) receptor may be effective inhibitors of SARS-CoV-2 cell entry. Here, we screened 512 pure compounds derived from natural products using a high-throughput RBD/ACE2 binding assay and identified (–)-hopeaphenol, a resveratrol tetramer, in addition to vatalbinoside A and vaticanol B, as potent and selective inhibitors of RBD/ACE2 binding and viral entry. For example, (–)-hopeaphenol disrupted RBD/ACE2 binding with a 50% inhibitory concentration (IC_50_) of 0.11 μM, in contrast to an IC_50_ of 28.3 μM against the unrelated host ligand/receptor binding pair PD-1/PD-L1 (selectivity index, 257.3). When assessed against the USA-WA1/2020 variant, (–)-hopeaphenol also inhibited entry of a VSVΔG-GFP reporter pseudovirus expressing SARS-CoV-2 spike into ACE2-expressing Vero-E6 cells and *in vitro* replication of infectious virus in cytopathic effect and yield reduction assays (50% effective concentrations [EC_50_s], 10.2 to 23.4 μM) without cytotoxicity and approaching the activities of the control antiviral remdesivir (EC_50_s, 1.0 to 7.3 μM). Notably, (–)-hopeaphenol also inhibited two emerging variants of concern, B.1.1.7/Alpha and B.1.351/Beta in both viral and spike-containing pseudovirus assays with similar or improved activities over the USA-WA1/2020 variant. These results identify (–)-hopeaphenol and related stilbenoid analogues as potent and selective inhibitors of viral entry across multiple SARS-CoV-2 variants of concern.

## INTRODUCTION

Severe acute respiratory syndrome coronavirus 2 (SARS-CoV-2) is the causative agent of coronavirus disease 2019 (COVID-19). Since crossing into humans in late 2019, SARS-CoV-2 has continued to cause substantial human morbidity and mortality worldwide. While SARS-CoV-2 vaccines are in development, with several approved for use, access to these vaccines remains limited, particularly in low- and middle-income countries. Moreover, vaccine hesitancy and ongoing mutation of SARS-CoV-2 increase the risk of resistance to vaccine-induced immune responses. Importantly, a significant gap in our therapeutic arsenal exists, as no outpatient chemotherapy strategy is currently licensed to prevent infection or to combat mild to severe COVID-19. For example, while chemotherapy against the RNA polymerase and neutralizing antibody infusions are available, they depend on access to patient infusion resources. Thus, broadly accessible SARS-CoV-2 antivirals to treat infections irrespective of hospitalization are urgently needed to complement ongoing vaccination efforts.

One attractive therapeutic target of SARS-CoV-2 replication is its binding and entry into host cells, which are induced by the trimeric viral spike glycoprotein (1). A primary cellular receptor of SARS-CoV-2 entry is the angiotensin-converting enzyme II (ACE2) protein. Viral entry is mediated by the receptor biding domain (RBD) of the S1 segment of spike, which directly interacts with ACE2, while S2 mediates membrane fusion (2, 3). Following RBD/ACE2 binding, SARS-CoV-2 gains entry to host cells through both an endosomal, clathrin-dependent pathway and a clathrin-independent pathway which involves spike protein cleavage by furin, transmembrane serine protease 2 (TMPRSS2), and other host proteases (1, 2). Antagonism of the RBD/ACE2 interaction would therefore be expected to block SARS-CoV-2 entry and replication.

Since its worldwide spread in early 2020, SARS-CoV-2 variants have acquired mutations within spike that enhance binding to ACE2 and increase infectivity, as first detected by the emergence of a D614G mutation which has since become dominant (4). More recently, additional variants have emerged with further spike sequence divergence; these include B.1.1.7/Alpha, which originated in the United Kingdom, and B.1.351/Beta (also called 501Y.V2) from South Africa (5). The B.1.1.7 variant contains an additional N501Y mutation within the RBD that causes reduced antibody neutralization by both convalescent and vaccine sera *in vitro* (6). Concerningly, the B.1.351 variant, which along with N501Y has also acquired mutations E484K and K417N within the RBD, has further reduced susceptibility to, or enhanced escape from, neutralizing antibodies and sera from convalescent and vaccine-treated patients and immunized mice (7, 8). As these observations raise concerns about the emergence of variants that become resistant to vaccine-induced immune responses, additional countermeasures with the potential to target emerging SARS-CoV-2 variants are essential.

**FIG 1:**
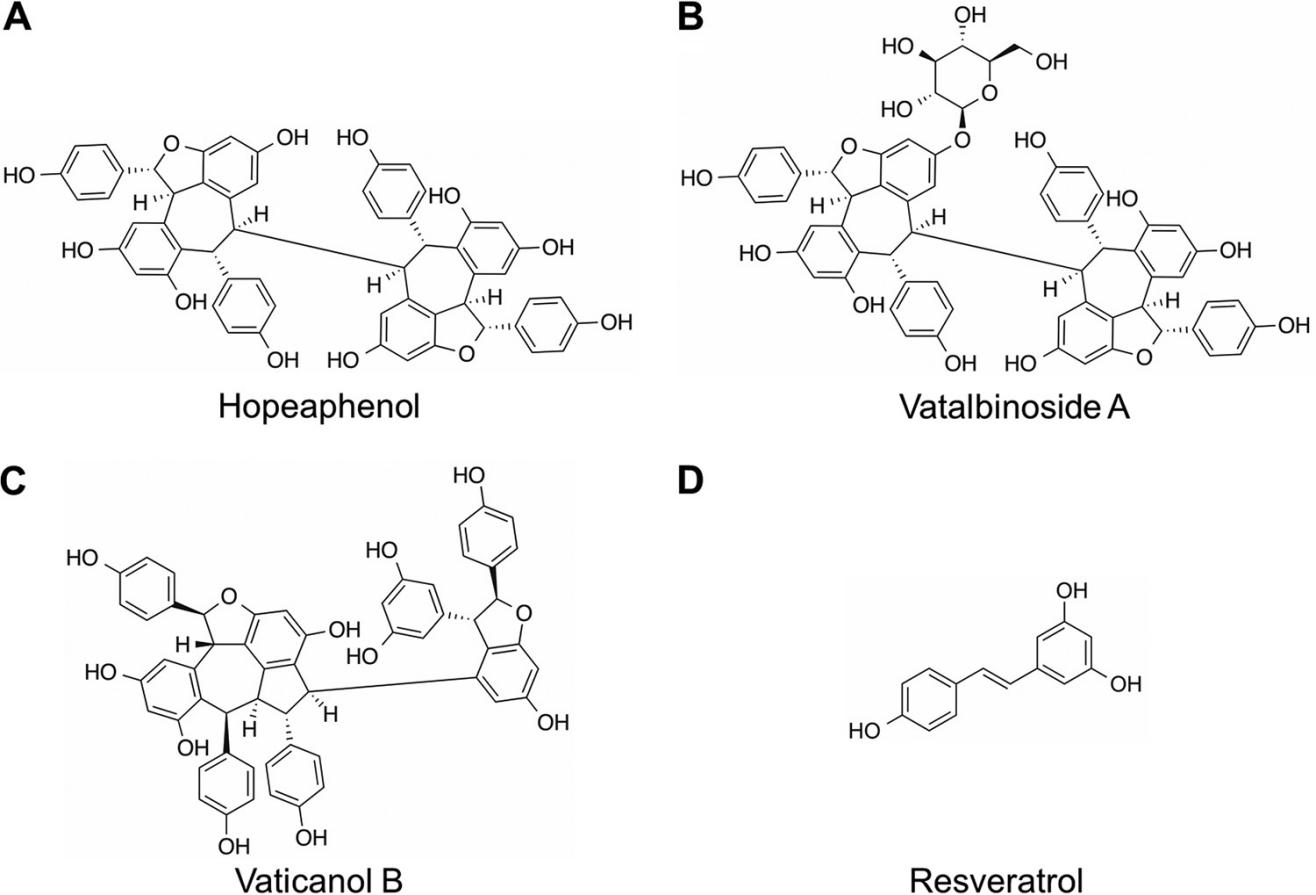
Chemical structures of (–)-hopeaphenol (A), vatalbinoside A (B), vaticanol B (C), and resveratrol (D).

Pure compounds derived from natural products are a rich source of antivirals, including against coronaviruses (9). However, outside computational studies, few natural products to date have been demonstrated to substantially reduce SARS-CoV-2 replication (10). To identify natural product compounds that may inhibit entry across multiple SARS-CoV-2 variants, we developed an AlphaScreen-based RBD/ACE2 interaction assay to screen a pure compound library containing 512 natural products and derivatives sourced predominantly from plants, mushrooms, and marine invertebrates of Australia, Papua New Guinea, and neighboring regions (11, 12). The top hits from this screen, which included the plant stilbenoids (–)-hopeaphenol, vatalbinoside A, and vaticanol B (Fig. 1), were then assessed for *in vitro* mechanisms of action using SARS-CoV-2 pseudoviruses and antiviral activity against infectious SARS-CoV-2 variants encompassing parental and B.1.1.7 and B.1.351 variants.

## RESULTS

### Stilbenoids selectively inhibit the SARS-CoV-2 spike RBD/host ACE2 protein interaction.

**FIG 2:**
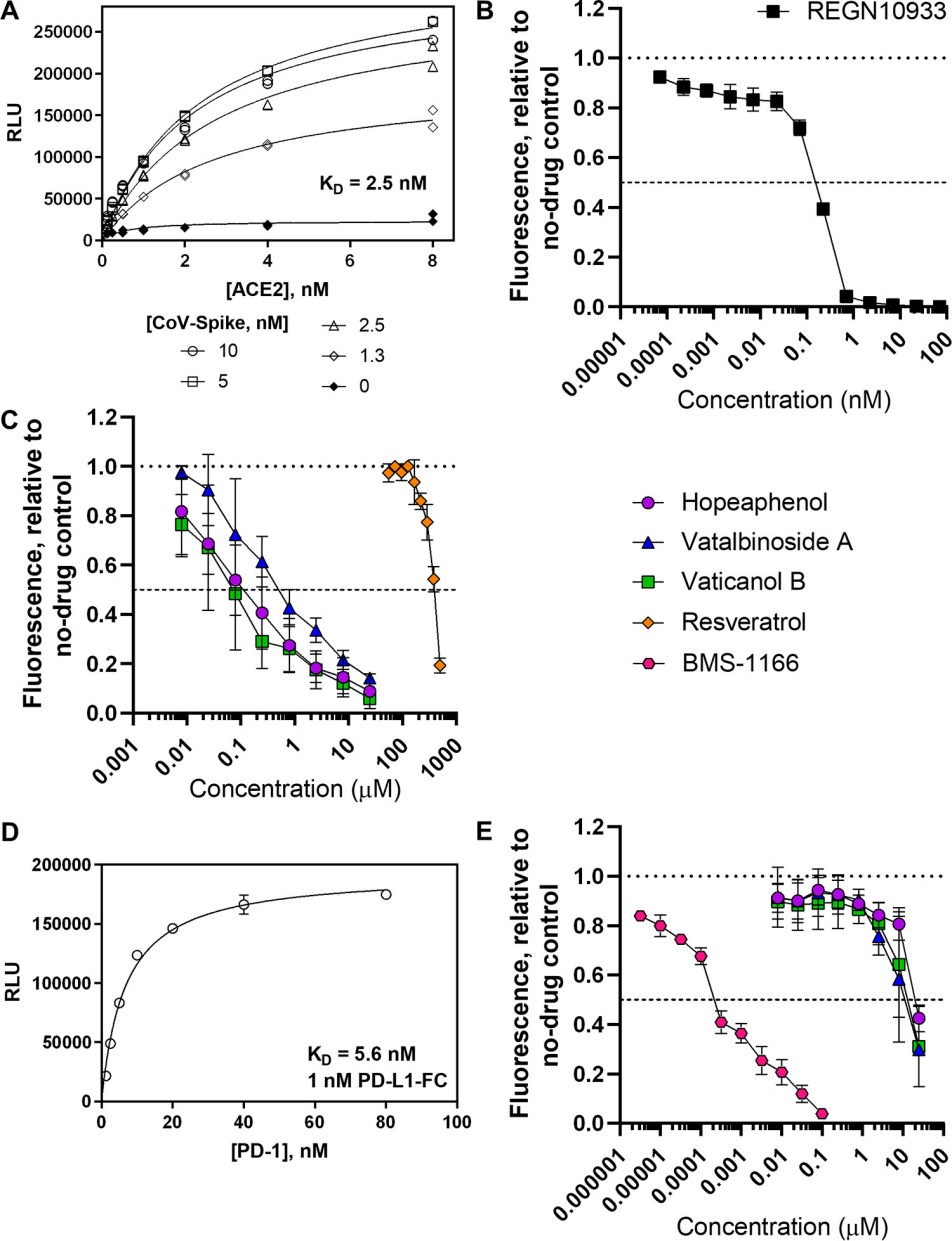
Identification of stilbenoids as spike-ACE2 inhibitors by AlphaScreen. (A) Demonstration of AlphaScreen-based luminescence due to interactions of His-tagged spike RBD (from USA-WA1/2020) and Fc-tagged ACE2 peptides. (B) Dose-response curve of REGN10933 on luminescence due to disruption of RBD/ACE2 interactions. (C) Dose-response curves of stilbenoids and resveratrol on luminescence inhibition due to disruption of RBD/ACE2 interactions. (D) Demonstration of AlphaScreen-based luminescence due to interactions of His-tagged PD-1 and Fc-tagged PD-L1 peptides. (E) Dose-response curves of stilbenoids and control inhibitor BMS-1166 on luminescence inhibition due to disruption of PD-1/PD-L1 interactions. Results in panels B, C, and E, denote the mean ± SEM from at least 3 independent experiments.

To identify potential inhibitors of SARS-CoV-2 entry, we used AlphaScreen technology (13) to develop a high-throughput, 384-well, plate-based assay to monitor the interaction of SARS-CoV-2 Spike RBD with host ACE2 (Fig. 2A). Briefly, a SARS-CoV-2 RBD protein derived from USA-WA1/2020 and containing a C-terminal His tag, in addition to a full-length ACE2 peptide with a C-terminal Fc tag, were prebound to respective acceptor and donor beads and coincubated for 3 h at room temperature. When a ligand/receptor binding event occurs, excitation at 680 nm results in a singlet oxygen transfer between donor and receptor beads, which results in luminescence at 615 nm. Compounds that inhibit binding of RBD to ACE2 should therefore inhibit luminescence. In the absence of compounds, we observed that luminescence was highly dependent on concentrations of both RBD and ACE2 (Fig. 2A) with a >200-fold signal to noise ratio and high reproducibility across experiments (Z′ > 0.8) (14). Further, when assessing the clinically approved neutralizing antibody REGN10933 (casirivimab), which acts by targeting spike RBD (15), we found a dose-dependent inhibition across 4 independent experiments, with a calculated average 50% inhibitory concentration (IC_50_) of 0.18 nM (Fig. 2B; Table 1).

**TABLE 1:** Summary of total stilbenoid and control compound bioactivities in AlphaScreen and pseudovirus assays

Compound	IC_50_ for:				Infected cells at 50 μM (%)		
	Spike/ACE2 AlphaScreen	PD-1/PD-L1 AlphaScreen (μM)	Selectivity index	M^pro^ activity (μM)	VSVΔG-S-GFP pseudovirus		
					WA1/2020	B.1.1.7	B.1.351
Hopeaphenol	0.11 μM	28.3	257.3	42.5	28.2	34.1	20.4
Vatalbinoside A	0.24 μM	22.3	92.9	68.7	40.4	43.8	41.1
Vaticanol B	0.067 μM	16.6	247.8	47.6	83.7	37.7	72.3
Resveratrol	>100 μM				96.3		
REGN10933	0.18 nM						
BMS-1166	>100 μM	0.0040					
GC-376				0.0052			

Using this assay, we then screened 512 pure compounds obtained from natural products and semisynthetic derivatives available from Compounds Australia at Griffith University at 3 μM, where 7 (1.4%) inhibited >75% of fluorescence observed in the absence of compounds. Activities of the top compounds were then assessed for dose-response profiles, where the three most active hits were a series of stilbenoid resveratrol tetramers, (–)-hopeaphenol, vatalbinoside A, and vaticanol B (Fig. 1 and 2C). These stilbenoids, exemplified by hopeaphenol, are tetramers of resveratrol available from multiple plant sources (16). Using the RBD/ACE2 AlphaScreen assay described above, dose-response profiles from 5 independent experiments were obtained to calculate IC_50_s of 0.11, 0.24, and 0.067 μM for hopeaphenol, vatalbinoside A, and vaticanol B, respectively (Table 1). In contrast, almost no activity by the resveratrol monomer was observed in this assay (IC*50* > 100 μM; Fig. 2C), indicating that inhibition requires a multimeric structure.

To confirm the selectivity of hopeaphenol and analogues to disrupt the RBD/ACE2 interaction, we next assessed their ability to interfere with the unrelated host PD-1/PD-L1 ligand/receptor pair using a comparable and previously described experimental approach (17), where we observed a >100-fold signal to noise ratio and Z′ of >0.75 (Fig. 2D). In this assay, the control PD-1/PD-L1 antagonist BMS-1166 (18) disrupted bead proximity-based fluorescence with an IC_50_ of 0.0040 μM (Fig. 2E) but had no activity against the RBD/ACE2 interaction (IC_50_ > 100 μM; data not shown). Conversely, hopeaphenol, vatalbinoside A, and vaticanol B were all substantially less effective in disrupting PD-1/PD-L1, with IC_50_s of 28.3, 23.3, and 16.6 μM, respectively (Fig. 2E; Table 1). From these two assays, the selectivity indices of hopeaphenol, vatalbinoside A, and vaticanol B (i.e., IC_50_ [PD-1/PD-L1]/IC_50_ [RBD/ACE2]) were calculated to be 257.3, 92.9, and 247.8, respectively, indicating high selectivity of these compounds for disrupting the viral RBD/host ACE2 interaction over an unrelated host ligand/receptor pair.

### Stilbenoids are weak inhibitors of viral main protease.

**FIG 3:**
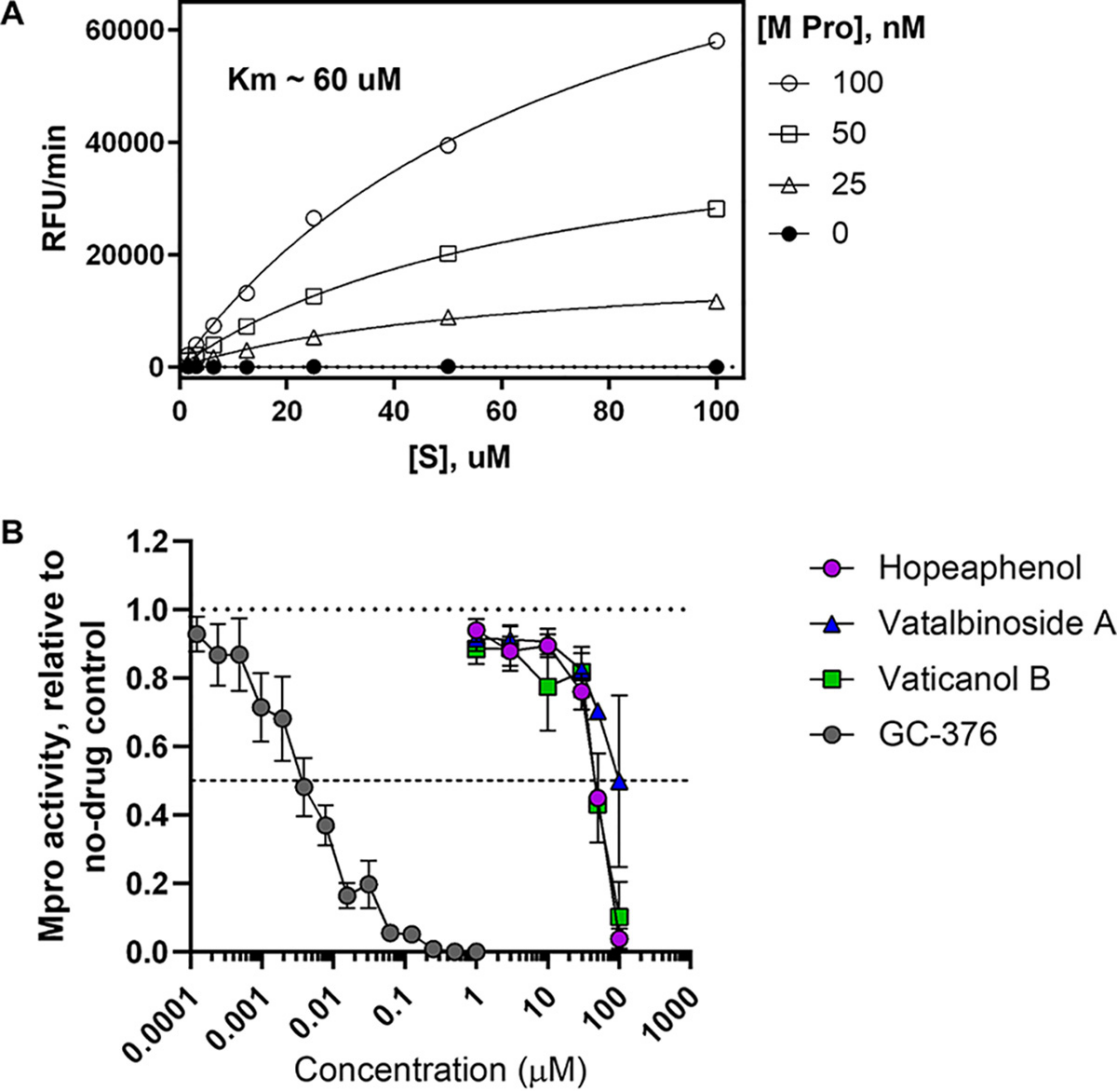
Effects of stilbenoids on inhibition of SARS-CoV-2 M^pro^ activity. (A) Demonstration of recombinant M^pro^ enzymatic activity on a fluorescence resonance energy transfer (FRET)-based fluorogenic peptide substrate. (B) Dose-response curves of stilbenoids and control inhibitor GC-376 on M^pro^ enzymatic activity. Results in panel B denote the mean ± SEM from at least 3 independent experiments.

A recent high-throughput virtual screening study proposed that hopeaphenol may act as an inhibitor of the SARS-CoV-2 main protease (M^pro^) by interfering with its active site (19), thereby raising the possibility of hopeaphenol acting on multiple viral targets. To test this possibility, we also developed an M^pro^ enzymatic assay using an M^pro^ peptide substrate resembling those described previously (20), where a C-terminal 5-((2-aminoethyl)amino)naphthalene-1-sulfonic acid (EDANS) fluorescent tag is quenched by an N-terminal 4-([4-(dimethylamino)phenyl]azo)benzoic acid (DABCYL) tag. Following incubation with recombinant M^pro^, the cleaved substrate affords separation of the EDANS tag from the DABCYL quencher and detection of fluorescence at 490 nm. Compounds that inhibit M^pro^ activity are therefore expected to inhibit fluorescence. This assay also exhibited a >10-fold signal to noise ratio and Z′ of >0.6 (Fig. 3A) and was adaptable to a 384-well screening format.

Using this assay, we observed that the control M^pro^ inhibitor GC-376 blocked enzymatic activity with an IC_50_ of 0.0052 μM, consistent with previous observations (Fig. 3B) (20, 21). In contrast, we observed that hopeaphenol, vatalbinoside A, and vaticanol B inhibited M^pro^ activity with IC_50_s of 42.5, 68.7, and 47.6 μM, respectively (Fig. 3B; Table 1), suggesting that these stilbenoids, while potentially capable of targeting M^pro^, are to a first approximation more effective against RBD binding to ACE2.

### Stilbenoids inhibit SARS-CoV-2 spike-dependent viral entry.

**FIG 4:**
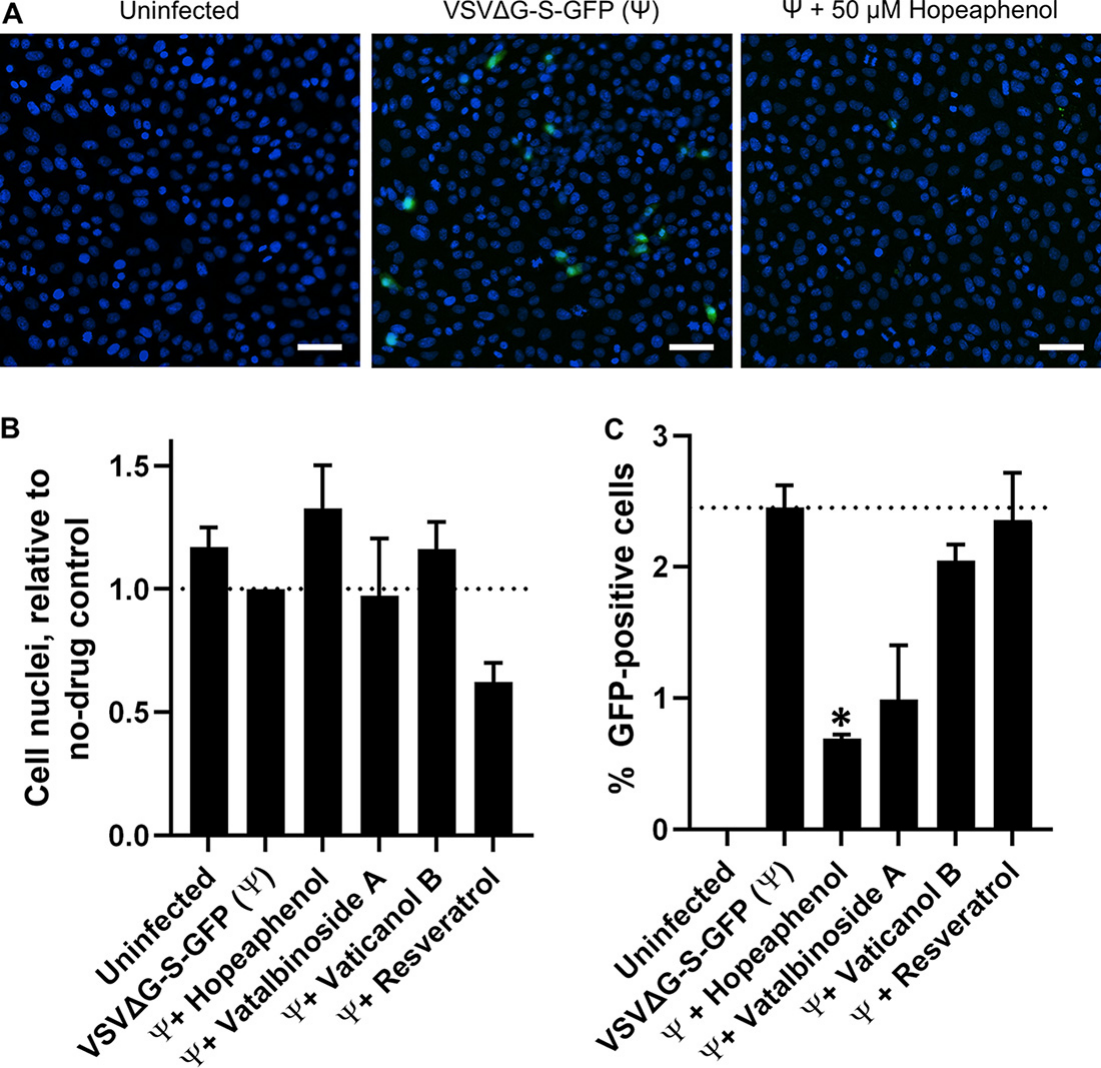
Effects of stilbenoids on *in vitro* pseudovirus entry. (A) Representative images of uninfected Vero-E6 cells (left) or cells infected with VSVΔG-S-GFP pseudovirus containing SARS-CoV-2 spike (USA-WA1/2020) in the absence (center) or presence of 50 μM hopeaphenol (right). Blue denotes Hoechst-stained cell nuclei, and green indicates GFP-positive infected cells. Scale bar = 100 μm. (B) Average number of total cell nuclei per cell field as counted by high-content imaging. (C) Percentage of GFP-positive (i.e., pseudovirus-infected) cells as measured by high-content imaging in the presence of stilbenoids. Results in panels B and C denote the mean ± SEM from 3 independent experiments. *, *P* < 0.05; ratio paired *t* test.

To assess whether hopeaphenol and analogues inhibit viral entry within a cellular context, we generated a single-cycle pseudovirus consisting of a vesicular stomatitis virus (VSV) backbone lacking the G fusion protein and expressing SARS-CoV-2 spike protein and green fluorescent protein (GFP) reporter (VSVΔG-S-GFP) (22). In our initial experiments, we generated pseudovirus with spike sequence corresponding to the USA-WA1/2020 variant. Pseudovirus was then incubated with cells in the presence or absence of stilbenoids in a 384-well format in duplicate. High-content imaging was then used to count the total live and infected cells in each culture across 15 nonoverlapping images per well, as determined by Hoechst-stained nuclei and cellular GFP fluorescence, respectively. This approach allowed us to image approximately 12,000 cells per well (see Table S1 in the supplemental material). Consistent with previous observations (23, 24), VSVΔG-S-GFP pseudovirus infected ACE2-expressing cells such as Vero-E6 (Fig. 4A) (25) but not cell lines lacking ACE2, such as BHK-21 cells (data not shown). In this assay, we observed an average of 2.4 ± 0.2% GFP-positive cells (mean ± standard error of the mean [SEM]) across 3 independent experiments following 24-h incubation with pseudovirus and 0.1% dimethyl sulfoxide (DMSO) vehicle control (Fig. 4A; Table S1) with no major changes in the number of cell nuclei relative to uninfected cells (Fig. 4B). Additionally, no major changes in total cell nuclei were observed in the presence of up to 50 μM any compound, indicating no overt effects on cell viability, with the exception of 50 μM resveratrol, which resulted in cultures with 60.3 ± 7.0% of nuclei observed in untreated, infected cells (Fig. 4B; Table S1).

However, when Vero-E6 cells were infected with pseudovirus in the presence of 50 μM hopeaphenol, the number of GFP-positive cells was reduced, with only 28.2 ± 1.2% of infected cells observed compared to infected cells treated with 0.1% DMSO (*P* = 3.5 × 10^−3^ versus cells treated with no drug; Fig. 4A and C; Table 1; Table S1). Similar results were observed when infected cells were cotreated with 50 μM vatalbinoside A, where only 40.4 ± 16.7% of GFP-positive cells were observed relative to infected, vehicle-treated cells, although this comparison did not reach statistical significance compared to untreated, infected cells (Fig. 4C). In contrast, 50 μM vaticanol B resulted in GFP expression in 83.7 ± 4.9% of cells, indicating that the potent anti-RBD/ACE2 activity observed by AlphaScreen assay was not reproduced in the pseudotype assay. As expected, 50 μM resveratrol had no effect on GFP-positive cells (96.3 ± 14.7% infected cells relative to infected, vehicle-treated cells; Fig. 4C). However, no compound inhibited GFP expression when incubated with infected cells at 15 μM (data not shown). Taken together, these results indicate that at least a subset of stilbenoids can inhibit entry of pseudoviruses expressing SARS-CoV-2 spike protein *in vitro*, consistent with AlphaScreen assay results, although this occurs at much higher concentrations.

### Stilbenoids inhibit infectious SARS-CoV-2 replication.

**FIG 5:**
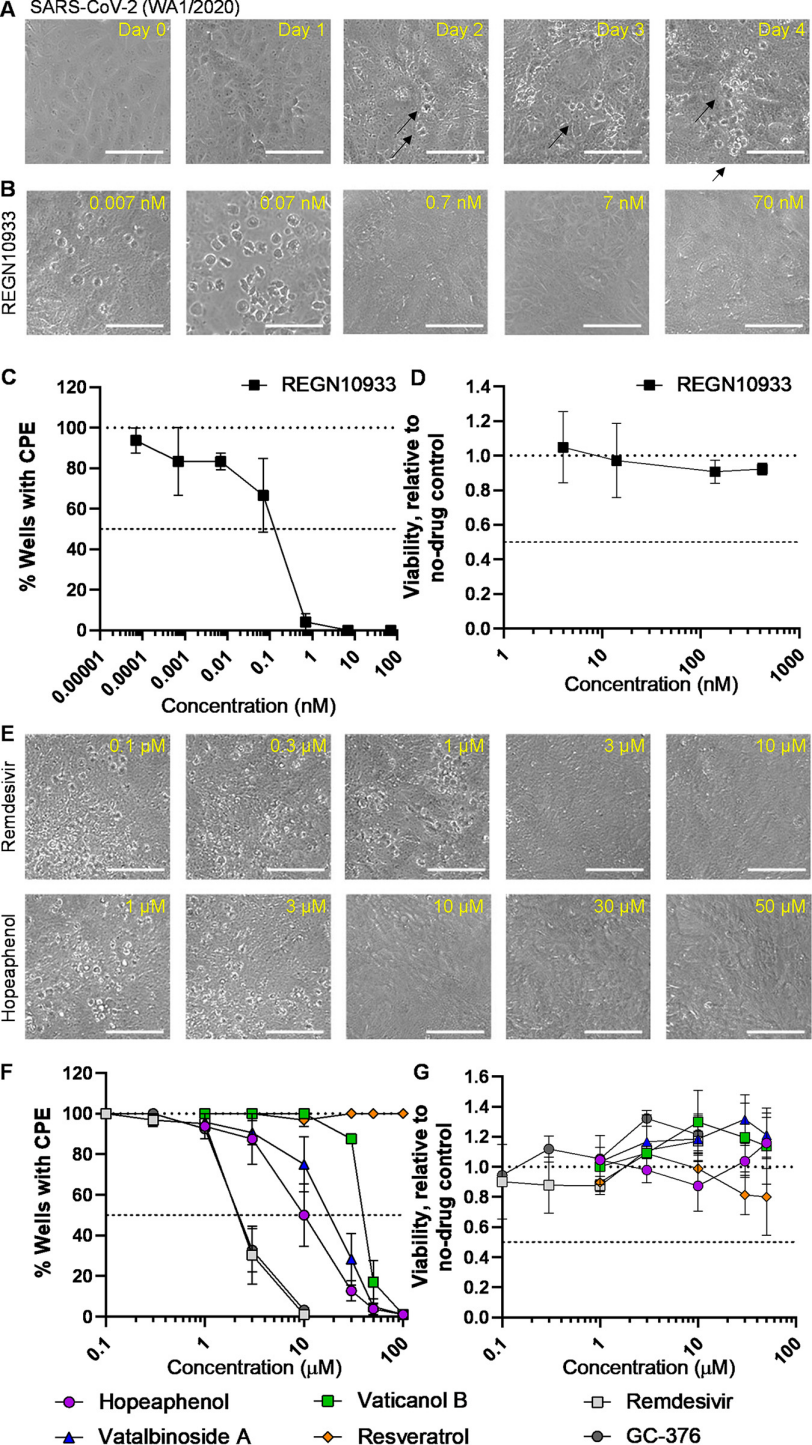
Effects of stilbenoids on infectious SARS-CoV-2 replication *in vitro* using a CPE scoring-based assay. (A) Representative images of Vero-E6 cells infected with 50× TCID_50_ of SARS-CoV-2 (USA-WA1/2020 variant) at 0 to 4 days postinfection. Arrows denote examples of CPE. (B) Representative images of infected cells in the presence of REGN10933 at the stated concentrations. (C) Dose-response curve of REGN10933 on viral replication in Vero-E6 cells after 4 days of infection. (D) Dose-response curve of REGN10933 on cell viability in uninfected Vero-E6 cells after 4 days of incubation. (E) Representative images of infected cells in the presence of remdesivir (top) and hopeaphenol (bottom) after 4 days of incubation at the stated concentrations. (F) Dose-response curves of stilbenoids, resveratrol, and remdesivir and GC-376 controls on viral replication in Vero-E6 cells after 4 days of infection. (G) Dose-response curves of compounds on cell viability in uninfected Vero-E6 after 4 days of incubation. In panels A, B, and E, scale bars = 100 μm. Results in panels C, D, F, and G denote the mean ± SEM from at least 3 independent experiments.

To assess the cellular antiviral activity of hopeaphenol and analogues, we first used a cytopathic effect (CPE) scoring-based assay with infectious virus in Vero-E6 cells (26, 27). Briefly, Vero-E6 cells were treated with agents for 2 h in 8-fold replicates in a 96-well format before infection with a 50× median tissue culture infectious dose (TCID_50_) of SARS-CoV-2 (USA-WA1/2020 variant). Cells were then incubated for 4 days with daily scoring of CPE across all wells by a user blinded to the experimental conditions. Using this approach, we observed the presence of CPE by 2 days postinfection, as characterized by extensive cell rounding and cellular debris that were observable by light microscopy (Fig. 5A, arrows). By 4 days postinfection, this CPE was observable across the cell culture and clearly distinguishable from uninfected cell controls (Fig. 5A). When low to subnanomolar concentrations of REGN10933 were added 2 h before infection, CPE was inhibited in these cultures after 4 days (Fig. 5B), with a calculated average 50% effective concentration (EC_50_) of 0.16 nM across 3 independent experiments (Fig. 5C; Table 2). We also observed no evidence of cytotoxicity due to REGN10933 treatment, as measured by resazurin staining following 4 days of treatment of uninfected Vero-E6 cells (Fig. 5D).

**TABLE 2:** Summary of total stilbenoid and control compound bioactivities in infectious SARS-CoV-2 assays

Compound	EC_50_ for CPE scoring-based assay for:			CC_50_ cell viability (μM)	EC_50_: viability restoration in infected cells for:			EC_50_: yield reduction assay for:		
	WA1/2020	B.1.1.7	B.1.351		WA1/2020	B.1.1.7	B.1.351	WA1/2020	B.1.1.7	B.1.351
Hopeaphenol	10.2 μM	14.8 μM	2.3 μM	>100	23.4 μM	7.8 μM	7.5 μM	13.5 μM	11.4 μM	8.8 μM
Vatalbinoside A	13.8 μM	4.0 μM	3.6 μM	>100	18.8 μM	>50 μM	11.3 μM	19.1 μM		
Vaticanol B	37.0 μM	32.4 μM	25.3 μM	>100	>50 μM	>50 μM	>50 μM	>50 μM		
Resveratrol	>100 μM			∼100	>50 μM					
REGN10933	0.16 nM	0.013 nM	9.2 nM		0.25 nM	0.10 nM	0.97 nM	0.17 nM	0.011 nM	2.2 nM
Remdesivir	2.5 μM	1.5 μM	1.4 μM	>10	7.3 μM	>10 μM	>10 μM	1.0 μM		
GC-376	3.9 μM			>10						

Similar to observations with REGN10933, when low micromolar concentrations of either the control nucleoside analog remdesivir or the M^pro^ inhibitor GC-376 (28, 29) were added 2 h before infection, CPE was also completely inhibited in these cultures after 4 days (Fig. 5E and F), with calculated EC_50_s of 2.5 and 3.9 μM for remdesivir and GC-376, respectively (Fig. 5C; Table 2). Moreover, comparable activity was observed in the presence of hopeaphenol (Fig. 5E, bottom), which blocked SARS-CoV-2 replication after 4 days with a calculated EC_50_ of 10.2 μM (Fig. 5F; Table 2). Notably, while similar antiviral activity was observed with vatalbinoside A (EC_50_, 13.8 μM), we observed substantially less activity with vaticanol B (EC_50_, 37.0 μM; Fig. 5F; Table 2), consistent with its reduced activity in pseudovirus assays (Fig. 4C). In contrast, no antiviral activity was observed with up to 100 μM resveratrol (Fig. 5F). No evidence of cytotoxicity was observed with these compounds (Fig. 5G).

**FIG 6:**
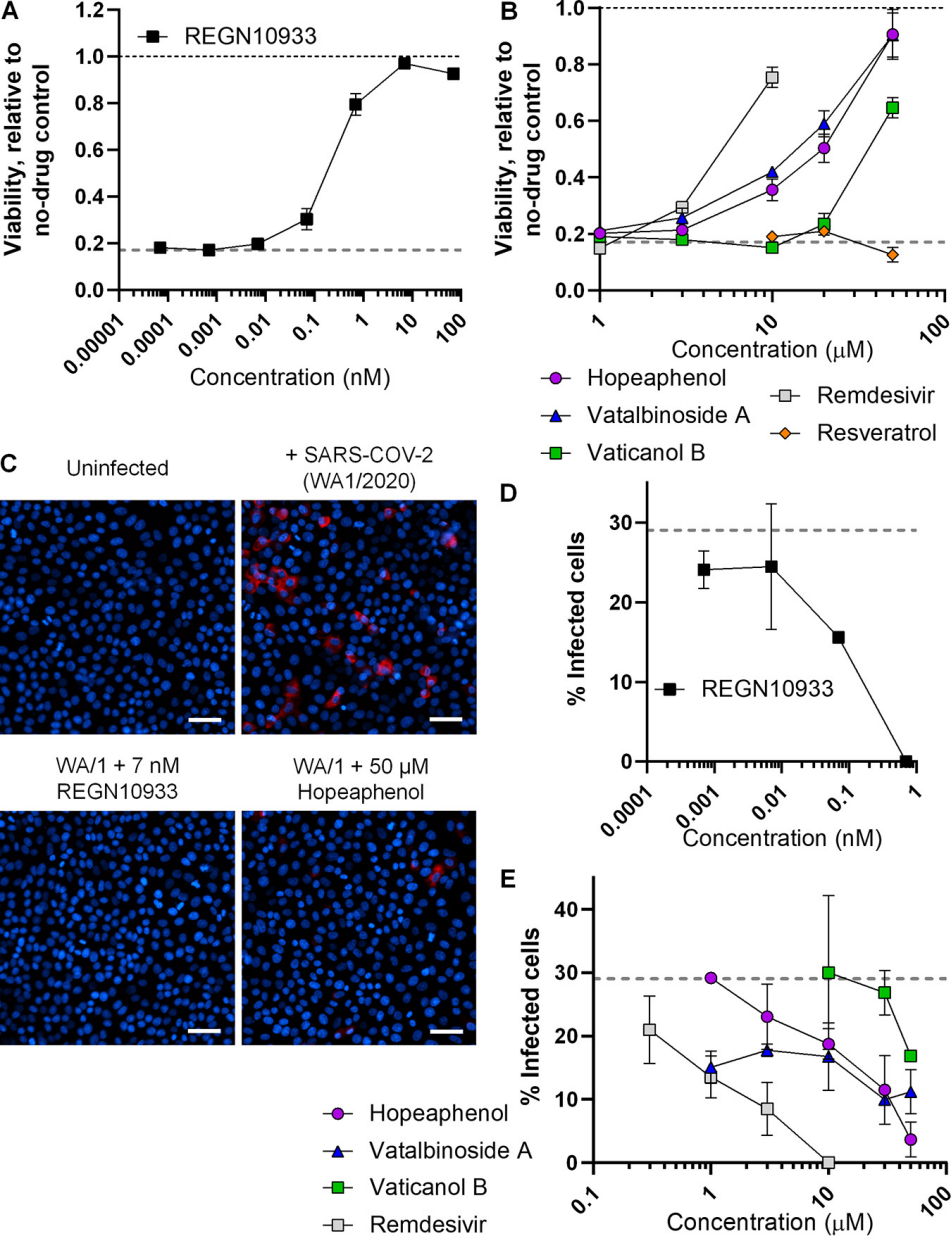
Quantitative effects of stilbenoids on infectious SARS-CoV-2 replication *in vitro*. (A) Dose-response curve of REGN10933 on cell viability in infected Vero-E6 cells after 4 days of infection with 150× TCID_50_ of SARS-CoV-2 (USA-WA1/2020 variant). (B) Dose-response curve of stilbenoids, resveratrol, and remdesivir on cell viability in infected Vero-E6 cells. The gray dotted lines in panels A and B indicate infected cell viability plus 0.1% DMSO vehicle control. Results in panels A and B represent the mean ± SEM from 3 independent experiments. (C) Representative images of Vero-E6 cells treated with 1:10 dilutions of supernatants from CPE scoring-based assays. Blue denotes Hoechst-stained cell nuclei, and red indicates SARS-CoV-2 nucleocapsid-positive infected cells. Scale bar = 50 μm. (D) Dose-response curve of REGN10933 on nucleocapsid-positive cells following supernatant treatments from CPE-scoring based assays. (E) Dose-response curves of stilbenoids and remdesivir on nucleocapsid-positive cells following supernatant treatments from CPE-scoring based assays. Dotted lines in panels D and E denote the percentage of nucleocapsid-positive cells following treatment with infected supernatants plus 0.1% DMSO. Results in panels D and E represent the mean ± SD from two independent experiments.

To confirm the antiviral activity of these compounds using more quantitative assessments, we next pretreated Vero-E6 cells with agents for 2 h in 8-fold replicates followed by infection with 150× TCID of USA-WA1/2020 virus. Four days following infection at this titer, we observed widespread CPE (data not shown), which when measured by resazurin staining, indicated an average of only 17.1 ± 0.2% cell viability relative to uninfected cells cultured in parallel across 3 independent experiments (*P* < 10^−4^; Fig. 6A; Table S2). When the same cells were treated with REGN10933, we observed a dose-dependent restoration of cell viability, consistent with abrogation of virus-induced CPE, with an EC_50_ of 0.25 nM (Table 2). Comparable but higher EC_50_s were observed in the presence of remdesivir (EC_50_, 7.3 μM), as well as hopeaphenol, vatalbinoside A, and vaticanol B (respective EC_50_s of 23.4, 18.8, and > 50 μM), while no evidence of restoration of cell viability occurred with up to 50 μM resveratrol (Fig. 6B; Table 2).

To determine whether reduction in CPE also corresponded to virus yield reduction, cell culture supernatants from 4 of 8 replicates from the CPE scoring-based assays described above were combined and applied to uninfected Vero-E6 cells at 1:10 dilution in a 384-well format in the absence of any additional chemical intervention. Following incubation with infected supernatants for 48 h, cells were fixed and probed for cellular SARS-CoV-2 nucleocapsid expression by immunostaining. Cell nuclei were counterstained with Hoechst, and high-content imaging was used to count total live and infected cells across 9 nonoverlapping images per well, which allowed us to image and score approximately 2,600 cells per well (Table S3). No nucleocapsid-positive cells were observed following treatment with uninfected culture supernatants (Fig. 6C). However, as shown in Fig. 6C, widespread nucleocapsid-positive cells (red) were observed following incubation with supernatants from USA-WA1/2020-infected cultures without drug treatment, which was determined to include an average of 29.0 ± 2.5% of all cells across 2 independent experiments (mean ± standard deviation [SD]). In contrast, we observed few nucleocapsid-positive cells following incubation with supernatants treated with 7 nM REGN10933 or 50 μM hopeaphenol (Fig. 6C; Table S3). We also observed dose-dependent inhibition of secondary viral infection from initial cultures treated with REGN10933 (EC_50_, 0.17 nM), as well as remdesivir (EC_50_, 1.0 μM), hopeaphenol (EC_50_, 13.5 μM), and vatalbinoside A (EC_50_, 19.1 μM), although less so for vaticanol B (EC_50_, >50 μM; Fig. 6E). Notably, as these EC_50_s correlated well with those observed in the CPE assays described above (Table 2), these observations suggest that the antiviral activity observed here is unlikely to be attributed to residual transfer and dilution of test agents.

Taken together, these results indicate that hopeaphenol and vatalbinoside A, and to a lesser extent vaticanol B, inhibit SARS-CoV-2 replication *in vitro* in three separate infection-based assays, with the activities of hopeaphenol and vatalbinoside A being at the same order of magnitude as those of the control SARS-CoV-2 antivirals remdesivir and/or GC-376.

### (–)-Hopeaphenol inhibits SARS-CoV-2 variants of concern with similar or improved activities.

**FIG 7:**
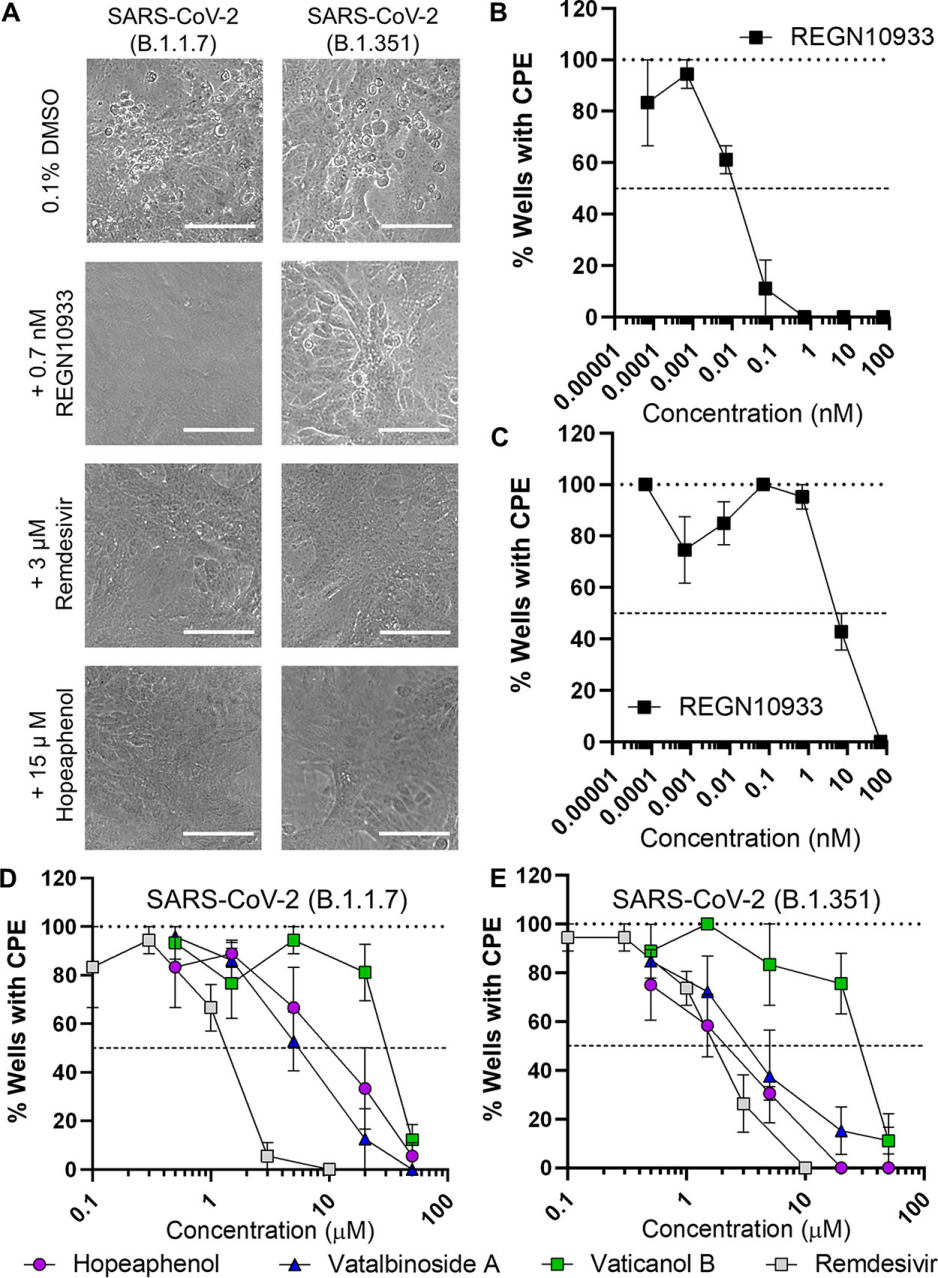
Effects of hopeaphenol and remdesivir on SARS-CoV-2 variant replication *in vitro* using a CPE scoring-based assay. (A) Representative images of Vero-E6 cells following 4 days of infection with either SARS-CoV-2 variant B.1.1.7 (left) or B.1.351 (right) in the presence of 0.1% DMSO vehicle control (top), 0.7 nM REGN10933 or 3 μM remdesivir (middle), or 15 μM hopeaphenol (bottom). Scale bar = 100 μm. (B and C) Dose-response curves of REGN10933 in Vero-E6 cells following 4 days of infection with SARS-CoV-2 variant B.1.1.7 (B) or B.1.351 (C). (D and E) Dose-response curves of stilbenoids and remdesivir in Vero-E6 cells following 4 days of infection with SARS-CoV-2 variant B.1.1.7 (D) or B.1.351 (E). In panels B and C, data are presented relative to infected cells treated with 0.1% DMSO vehicle control. Results in panels B to E denote the mean ± SEM from at least 3 independent experiments.

To determine if hopeaphenol maintained activity against emerging SARS-CoV-2 variants with accumulated mutations in the spike RBD, we first repeated the CPE scoring-based assay using two SARS-CoV-2 variants of concern, B.1.1.7 (England/204820464/2020; Alpha) and B.1.351 (KRISP-K005325/2020; Beta) (Fig. 7). Similar to our previous observations, infection of Vero-E6 cells with either strain resulted in detectable CPE across cultures after 4 days (Fig. 7A, top), which was completely abolished by pretreatment with 0.7 nM REGN10933 in cells infected with B.1.1.7 but required higher concentrations in cells infected with B.1.351 (Fig. 7A, middle). These results corresponded to EC_50_s of 0.013 and 9.2 nM in cells infected with B.1.1.7 or B.1.351, respectively (Fig. 7B and C; Table 2), consistent with previous reports indicating variable *in vitro* inhibitory activity for REGN10933 against SARS-CoV-2 variants (5). We also observed dose-dependent inhibition of these two variants by remdesivir, with calculated EC_50_s of 1.5 and 1.4 μM for B.1.1.7 and B.1.351 strains, respectively (Fig. 7D and E), which was in agreement with observations using USA-WA1/2020 virus (EC_50_, 2.5 μM; Table 2). Notably, 15 μM hopeaphenol also completely abrogated CPE by both variants (Fig. 7A, bottom). When assessed for dose-response profiles, we observed that B.1.1.7 was inhibited by hopeaphenol with an EC_50_ of 14.8 μM (Fig. 7D), which approximated hopeaphenol’s activity against USA-WA1/2020 (EC_50_, 10.2 μM; Table 2). In contrast, B.1.351 was inhibited by hopeaphenol with an EC_50_ of 2.3 μM (Fig. 7E), indicating 4.5-fold improved activity over USA-WA1/2020 (Table 2). Similar results were observed in the presence of vatalbinoside A, which inhibited B.1.1.7 and B.1.351 infection with EC_50_s of 4.0 and 3.6 μM, respectively, as well as vaticanol B, which inhibited infection with EC_50_s of 32.4 and 25.3 μM, respectively (Fig. 7D and E; Table 2).

**FIG 8:**
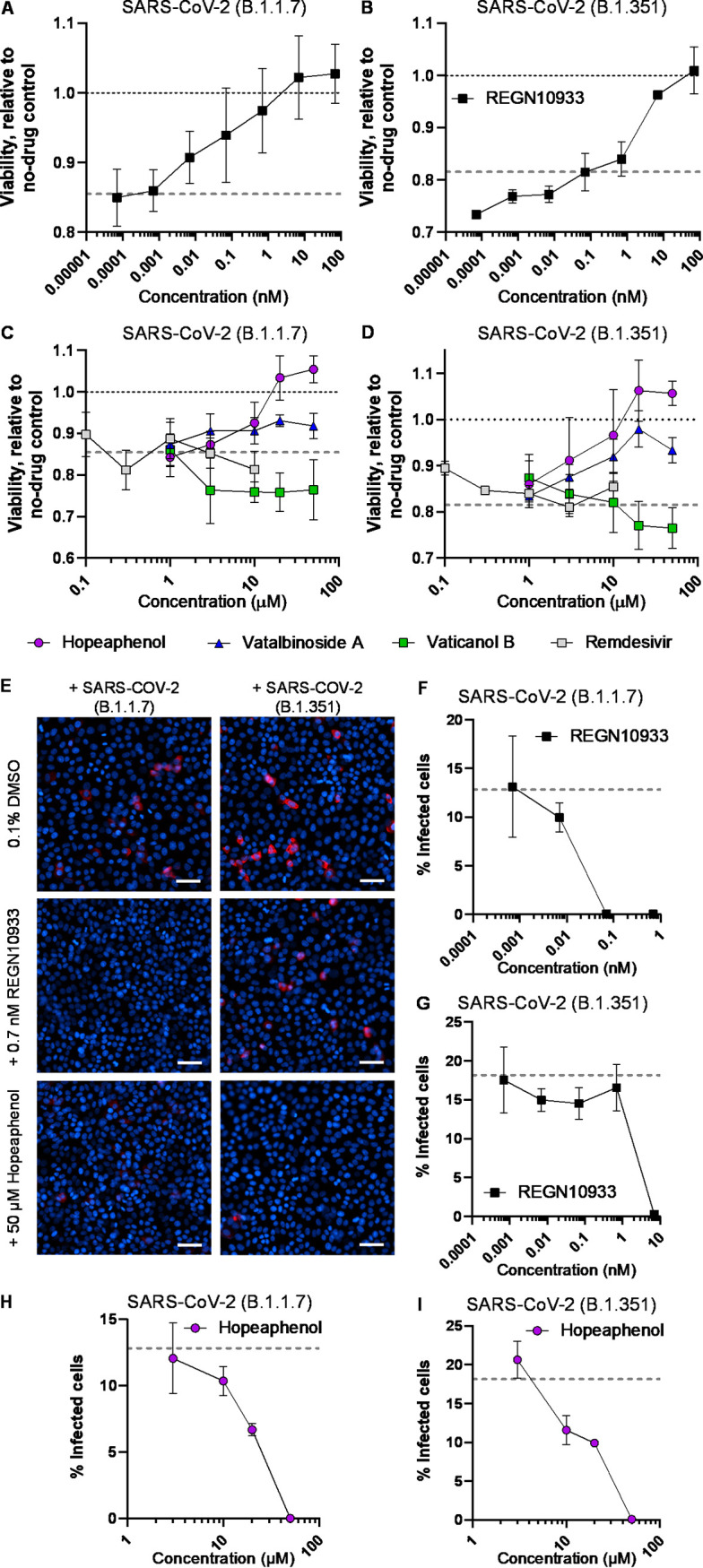
Quantitative effects of stilbenoids on infectious SARS-CoV-2 variant replication *in vitro*. (A and B) Dose-response curve of REGN10933 on cell viability in infected Vero-E6 cells after 4 days of infection with 150× TCID_50_ of SARS-CoV-2 variant B.1.1.7 (A) or B.1.351 (B). (C and D) Dose response curves of stilbenoids and remdesivir on cell viability in infected Vero-E6 cells after 4 days of infection with B.1.1.7 (C) or B.1.351 (D). The lower gray dotted lines in panels A to D indicate infected cell viability plus 0.1% DMSO vehicle control. Results in panels A to D denote the mean ± SEM from at least 3 independent experiments. (E) Representative images of Vero-E6 cells treated with 1:10 dilutions of supernatants from CPE scoring-based assays. Results are presented as described in Fig. 6C. Only single nucleocapsid-positive cells were detected in the presence of 50 μM hopeaphenol. (F and G) Dose-response curve of REGN10933 on nucleocapsid-positive cells following treatment from supernatants infected with B.1.1.7 (F) or B.1.351 (G). (H to I) Dose-response curves of hopeaphenol on nucleocapsid-positive cells following treatment from supernatants infected with B.1.1.7 (H) or B.1.351 (I). Dotted lines in panels F to I denote the percentage of nucleocapsid-positive cells following treatment with infected supernatants plus 0.1% DMSO. Results in panels F to I represent the mean ± SD from two independent experiments.

To confirm these findings, we next repeated the quantitative viability restoration assay described above. However, when cells were infected with 150× TCID_50_ of either B.1.1.7 or B.1.351, no significant reductions in viability were observed until 7 days postinfection, which resulted in an average across 3 independent experiments of 85.5 ± 1.7% viability in B.1.1.7-infected cells (*P* = 0.007) and 81.5 ± 2.8% viability in B.1.351-infected cells (*P* = 0.026) compared to uninfected cells tested in parallel (Fig. 8A to D; Table S2). The source of this reduced CPE by 150× TCID_50_ of B.1.1.7 and B.1.351 compared to USA-WA1/2020 is not immediately clear but could reflect more rapid adaptation of USA-WA1/2020 to Vero-E6 culture in these experiments. Regardless, treatment with REGN10933 was able to restore viability in both sets of infected cells with respective EC_50_s of 0.10 and 0.97 nM (Fig. 8A and B; Table 2). Hopeaphenol also restored viability in cells infected with B.1.1.7 or B.1.351, with respective EC_50_s of 7.8 and 7.5 μM (Fig. 8C and D; Table 2). In contrast, viability was only somewhat restored with vatalbinoside A treatment; for example, while we determined an EC_50_ of 11.3 μM in B.1.351-infected cells, no more than 51.2 ± 12.2% restored viability at 20 μM was observed in B.1.1.7-infected cells. Vaticanol B and remdesivir also did not restore viability at any concentration up to 50 or 10 μM, respectively (Fig. 8C and D; Table 2). These results suggest that remdesivir and vaticanol B, and to a lesser extent vatalbinoside A, do not maintain antiviral activity over 7 days’ culture in this context, perhaps due to reduced compound stability and/or the adaptation of viruses to the presence of compounds over this extended time period.

In virus yield reduction assays where B.1.1.7- and B.1.351-infected supernatants from CPE scoring-based assays were applied in 1:10 dilution to naive Vero-E6 cells and scored 48 h later for nucleocapsid protein by high-content imaging, we measured infection in 12.8 ± 1.1% of cells treated with B.1.1.7-infected supernatants and 18.1 ± 0.7% of cells treated with B.1.351-infected supernatants (Fig. 8E). We also observed dose-dependent inhibition from supernatants previously treated with REGN10933 (EC_50_s of 0.011 and 2.2 nM, respectively, for cells treated with supernatants from B.1.1.7 or B.1.351 infection REGN10933; Fig. 8F and G; Table 2). These observations also extended to infected supernatants previously treated with hopeaphenol, where EC_50_s of 11.4 and 8.8 μM were observed for supernatants from B.1.1.7- and B.1.351-infected cultures, respectively (Fig. 8H and I; Table 2). These results indicate that hopeaphenol inhibits replication of two SARS-CoV-2 variants across the three infection-based assays *in vitro*.

**FIG 9:**
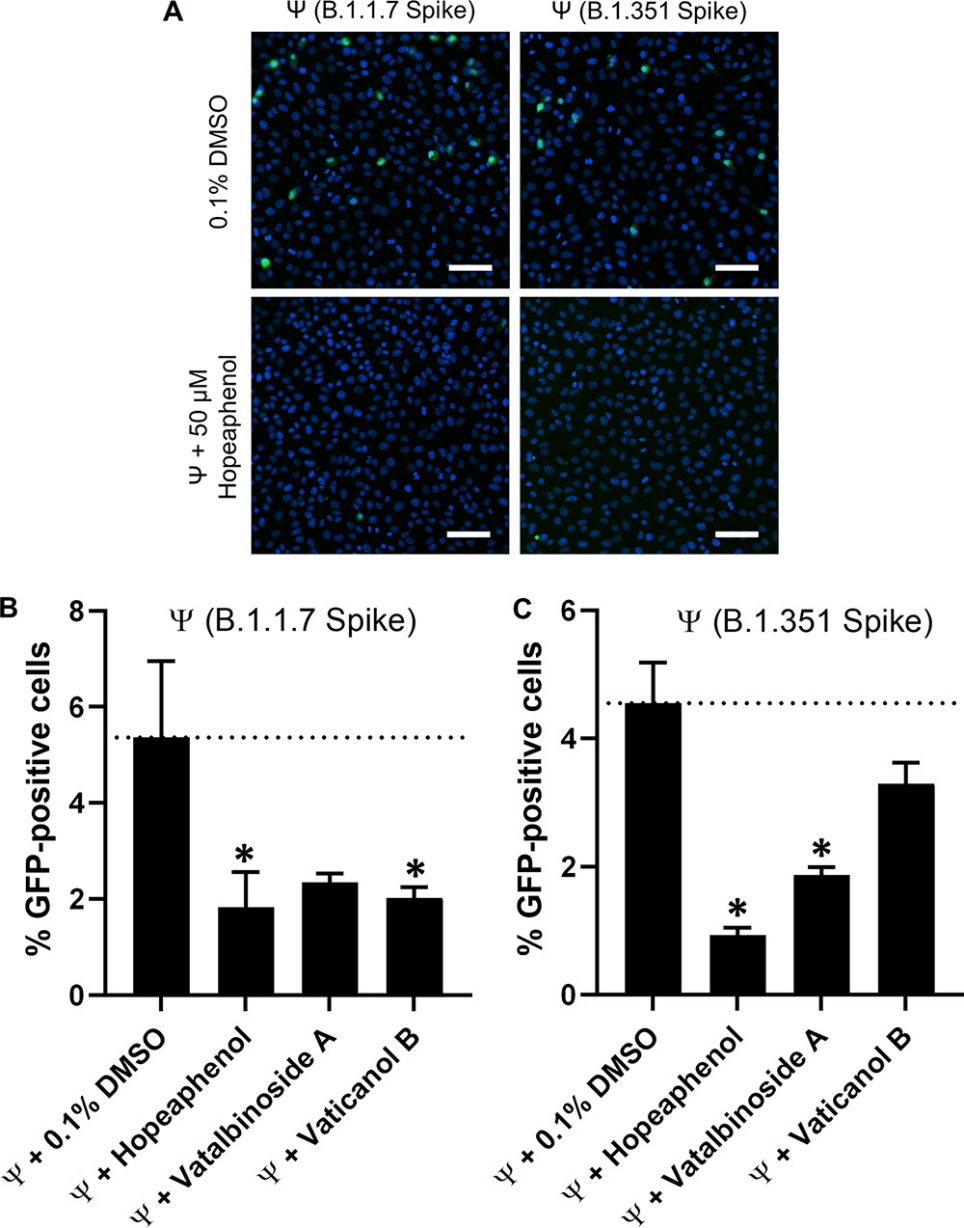
Effects of hopeaphenol on entry of pseudoviruses containing SARS-CoV-2 spike variants. (A) Representative images of Vero-E6 cells infected with VSVΔG-S-GFP pseudovirus containing SARS-CoV-2 spike from B.1.1.7 (left) or B.1.351 variants (right) in the presence of 0.1% DMSO (top) or 50 μM hopeaphenol (bottom). Images are organized as described in Fig. 4 (B and C) The percentage of B.1.1.7 (B) or B.1.351 (C) pseudovirus-infected cells in the presence of 0.1% DMSO or 50 μM stilbenoids. Results in panels B and C represent the mean ± SEM from at least 3 independent experiments. *, *P* < 0.05; ratio paired *t* test.

To confirm that the antiviral activities of stilbenoids against these variants of concern corresponded to inhibition of viral entry, we generated VSVΔG-S-GFP-based pseudoviruses containing B.1.1.7 or B.1.351 spike sequences and infected Vero-E6 cells in the absence or presence of 50 μM hopeaphenol, vatalbinoside, or vaticanol B (Fig. 9). Similar to previous observations (Fig. 4B), no major changes in total cell nuclei number were observed under any experimental condition (Table S1). Also, broadly consistent with previous observations with our original pseudovirus, we observed an average of 5.4 ± 1.6 and 4.6 ± 0.6% GFP-positive cells following 24-h incubation with pseudovirus containing B.1.1.7 or B.1.351 spike, respectively (Fig. 9A, top). Furthermore, infection of both pseudoviruses continued to be inhibited by 50 μM hopeaphenol (Fig. 9A, bottom). For example, pseudovirus containing B.1.1.7 spike was observed in only 34.1 ± 13.6% of cells relative to infected, vehicle-treated cells treated with 0.1% DMSO (*P* = 4 × 10^−4^; Fig. 9B; Table 1; Table S1), similar to results from pseudovirus plus USA-WA1/2020 spike (i.e., 28.2 ± 1.2%). Additionally, pseudovirus containing B.1.351 spike was present in only 20.4 ± 2.4% of infected, vehicle-treated cells compared to infected cells without 50 μM hopeaphenol treatment (*P* = 0.01; Fig. 9C). Cells infected with B.1.351 pseudovirus were reduced in the presence of 50 μM vatalbinoside A or vaticanol B, comparable to observations with USA-WA1/2020 pseudovirus (i.e., 41.1 ± 2.6 and 72.3 ± 7.3% of positive cells in no-drug, infected cultures; Fig. 9C). In contrast, vatalbinoside A and vaticanol B both inhibited B.1.1.7 pseudovirus infection at similar levels to what was observed for hopeaphenol (43.8 ± 3.4 and 37.7 ± 4.1%, respectively; Fig. 9B).

Taken together, these results indicate that hopeaphenol, and to a lesser extent vatalbinoside A and vaticanol B, inhibit both replication of infectious SARS-CoV-2 variants of concern *in vitro* and entry of pseudoviruses containing divergent SARS-CoV-2 spike sequences. In the case of hopeaphenol, its activities against B.1.1.7 and B.1.351 are similar or improved compared to activities against USA-WA1/2020.

## DISCUSSION

Antivirals that act across multiple SARS-CoV-2 variants are needed worldwide to supplement ongoing vaccine efforts, to provide therapeutic options for mild to severe SARS-CoV-2 infections irrespective of hospitalization, and to support COVID-19 treatment in resource-limited regions with poor access to intravenous antibody infusions. Here, we investigated a library of pure compounds containing 512 pure natural products and derivatives and identified three stilbenoids, exemplified by (–)-hopeaphenol, that disrupt the interaction of viral spike RBD with its host ACE2 receptor, block viral entry of spike-containing pseudoviruses, and antagonize infectious SARS-CoV-2 replication without cytotoxicity *in vitro*. Importantly, hopeaphenol also inhibits two emerging variants of concern (B.1.1.7 and B.1.351) which have acquired sequence variations that enhance SARS-CoV-2 infectivity and/or promote reduced susceptibility or enhanced escape from neutralizing antibodies. Although the concentrations of hopeaphenol needed to inhibit virus replication are much higher than those of REGN10933 antibody, active concentrations of hopeaphenol do approximate those of the small molecule antivirals remdesivir and GC-376. Hopeaphenol and other stilbenoid analogues are therefore promising leads for developing broad-spectrum SARS-CoV-2 entry inhibitors, potentially for use as monotherapies or in combination with antiviral leads against other viral targets, for example, to improve efficacy, reduce toxicities, and/or raise genetic barriers to potential viral resistance for clinical-stage nucleoside prodrugs and M^pro^ inhibitors compared to use as monotherapies (30, 31). These results also support studies to assess activity against additional variants of concern (i.e., B.1.617.2/Delta) as they continue to emerge and spread.

(–)-Hopeaphenol, vatalbinoside A, vaticanol B, and related stilbenoids and their stereoisomers have been isolated from a variety of plant sources, including *Hopea*, *Vitis*, *Shorea*, *Anisoptera*, and *Vatica* species, among others (32–40). These compounds have been reported to exhibit several *in vitro* properties, including antiproliferative (35, 36, 40, 41), antibacterial (through inhibition of the type III secretion system of Gram-negative bacteria) (33, 42), antifungal (43), anti-influenza and -herpes simplex virus (44, 45), and anti-inflammatory (37, 46) activities, among others. Notably, hopeaphenol is additionally reported to inhibit plasma triglyceride elevation in olive oil-treated mice and reduce plasma glucose in sucrose-loaded mice at 200 mg/kg (47, 48). It also exhibited hepatoprotective effects against lipopolysaccharide (LPS)-induced liver injury in mice at 100 mg/kg (37). These initial *in vivo* efficacy studies, which indicate tolerability at high concentrations, support near-term *in vivo* studies of anti-SARS-CoV-2 efficacy by (–)-hopeaphenol, although the concentrations of hopeaphenol that might be relevant *in vivo* compared to the *in vitro* concentrations identified here remain to be determined.

More recently, stilbenoids have been proposed as potential disruptors of SARS-CoV-2 spike protein with ACE2 in molecular docking studies (49). Another stilbenoid, kobophenol A, was also recently reported to inhibit binding of RBD with ACE2 (IC_50_, 1.8 μM) and SARS-CoV-2 replication (EC_50_, 71.6 μM) (50), and our data are consistent with these observations. However, we observed no antiviral activity by resveratrol at up to 100 μM, which contrasts with another recent study reporting an EC_50_ of 10.7 μM in SARS-CoV-2-infected Vero-E6 cells (BetaCov isolate) (51). However, this study measured supernatant viral RNA levels by quantitative PCR after 48 h of infection, so disparate results could reflect differences in sensitivity between quantitative PCR and the assays used here. Another consideration is that the three hit stilbenoid compounds, which are all polyphenolics, represent a structure class that has been given a PAINS (pan assay interference compounds) designation (52). Although we observed that these compounds selectively disrupted RBD/ACE2 binding over an unrelated PD-1/PD-L1 ligand/receptor pair, for example, with 257.3-fold selectivity for hopeaphenol, caution must still be exercised when considering these compounds for further therapeutic development. However, generation or isolation of stilbenoid analogues with potentially improved selectivity and assessment of chemical leads in primary cell models remain warranted.

While the three stilbenoids identified here selectively disrupted RBD/ACE2 interactions at submicromolar concentrations over an unrelated PD-1/PD-L1 ligand/receptor pair, inhibitory activity against spike-containing pseudoviruses occurred only at 50 μM, although low micromolar activity was generally observed for hopeaphenol and/or vatalbinoside A in infectious virus assays. These observations could partially reflect reduced activity against VSV-backbone pseudoviruses in particular and/or reduced stability of these stilbenoids *in vitro*. For example, reduced activity could be associated with assay-dependent structural differences in spike proteins, particularly with regard to pseudovirus assays. However, stilbenoids are also reported to be sensitive to oxidation due to the presence of the phenolic moieties and their ability to delocalize an unpaired electron (53). They are also unstable to factors including oxygen, heat, light, and pH changes (54, 55). Consistent with the potential for reduced stability, vaticanol B, while the most potent small molecule disruptor of RBD/ACE2 interactions by AlphaScreen, was less effective than hopeaphenol and vatalbinoside A in both pseudovirus and across infectious virus assays (Tables 1 and 2). Moreover, vatalbinoside A (and remdesivir) was also less active after 7 days of culture in virus restoration assays using SARS-CoV-2 variants, as opposed to hopeaphenol, which maintained activity (Table 2). Although more study is required, these observations suggest that hopeaphenol may have improved stability-dependent antiviral activity relative to other investigated stilbenoids and perhaps remdesivir.

A recent report also describes use of a virtual screening approach which identified hopeaphenol as a potential inhibitor of SARS-CoV-2 M^pro^ by interacting within its active site (19). In contrast, we observed only weak inhibitory activity of hopeaphenol and analogues against M^pro^ (e.g., IC_50_s of ∼40 to 70 μM, compared to 0.0052 μM for GC-376; Table 1). While our studies do not rule out modest inhibition of M^pro^ by hopeaphenol, this activity is unlikely to confer the primary antiviral activity observed *in vitro* (e.g., hopeaphenol EC_50_s of 2.3 to 23.4 μM in infectious viral assays; Table 2). Nevertheless, these combined results do raise the intriguing possibility of identifying stilbenoid derivatives that target both SARS-CoV-2 entry and M^pro^, which in turn may improve antiviral activity and/or reduce the risk of eventual viral drug resistance.

There are currently no licensed antivirals that reliably protect against COVID-19 in outpatient settings. Recent reports of SARS-CoV-2 variants with accumulated spike mutations and reduced susceptibility or escape from neutralizing sera from convalescent and vaccine-treated patients (7, 8) also raise the concern of emerging variants with resistance to preexisting immune responses. In contrast, we observe that hopeaphenol, despite acting as an inhibitor of spike-mediated viral entry, inhibits *in vitro* replication of both an early SARS-CoV-2 isolate (USA-WA1/2020) and two more recently emerging variants of concern (B.1.1.7 and B.1.351) with comparable or improved activity. These results suggest that spike mutations that promote vaccine-induced viral escape may be distinct from those that might arise from ongoing treatment with hopeaphenol and potentially other stilbenoid-based entry inhibitors. Although further studies are clearly needed, this possibility, in turn, raises the prospect of natural product-based entry inhibitors that function as effective antiviral countermeasures in the absence of available second-generation vaccines or therapies requiring inpatient administration.

## MATERIALS AND METHODS

### Chemical libraries and hit compounds.

The Davis Open Access Natural Product-Based Library consists of 512 distinct compounds, the majority (53%) of which are natural products obtained primarily from Australian fungal, plant, and marine invertebrate sources (11, 12), as well as semisynthetic natural product analogues (28%) and known commercial drugs or synthetic compounds inspired by natural products (19%). All compounds evaluated in this study were analyzed for purity prior to testing and shown to be >95% pure. Compounds were initially provided by Compounds Australia at Griffith University in 5-mM stock solutions dissolved in dimethyl sulfoxide (DMSO; Sigma-Aldrich, Sydney, Australia); as such, DMSO was used as the vehicle control in this study. The three hit compounds identified following library screening, which are all known stilbenoids (33), were resupplied as dry powders for confirmation studies and further biological evaluation.

### Cells, viruses, and reagents.

Vero-E6 cells were obtained from the American Tissue Culture Collection. Vero-E6 cells were cultured in D10+ medium (Dulbecco’s modified Eagle medium with 4.5 g/liter glucose and l-glutamine [Gibco, Gaithersburg, MD], 10% fetal bovine serum [Gemini Bio Products, West Sacramento, CA, USA], 100 U of penicillin/ml, and 100 μg of streptomycin/ml [Sigma-Aldrich, St. Louis, MO]) in a humidified incubator at 37°C and 5% CO_2_. BHK-21/WI-2 cells were purchased from Kerafast (Boston, MA, USA) and cultured in D5+ medium, which is identical to D10+ medium except for the addition of 5% fetal bovine serum.

The following reagent was deposited by the Centers for Disease Control and Prevention and obtained through BEI Resources, NIAID, NIH: SARS-related coronavirus 2, isolate USA-WA1/2020, NR-52281. The following reagents were obtained through BEI Resources, NIAID, NIH: SARS-related coronavirus 2, isolate hCoV-19/England/204820464/2020, NR-54000, contributed by Bassam Hallis, and SARS-related coronavirus 2, isolate hCov-19/South Africa/KRISP-K005325/2020, NR-54009, contributed by Alex Sigal and Tulio de Oliveira.

Remdesivir and resveratrol were purchased from Sigma-Aldrich. GC-376 was purchased from Selleckchem (Houston, TX, USA). REGN10933 was obtained from excess aliquot volumes at the Perelman School of Medicine which could not be used for patients. Isolation, structural confirmation, and purity of (–)-hopeaphenol, vatalbinoside A, and vaticanol B used in this study were reported previously (33).

### Protein-protein interaction assays.

SARS-CoV-2 spike-RBD binding to ACE2 was determined using AlphaScreen technology. First, 2 nM ACE2-Fc (Sino Biological, Chesterbrook, PA, USA) was incubated with 5 nM His-tagged SARS-CoV-2 spike-RBD (Sino Biological) in the presence of 5 μg/ml nickel chelate donor bead in a total of 10 μl of 20 mM Tris (pH 7.4), 150 mM KCl, and 0.05% CHAPS (3-[(3-cholamidopropyl)-dimethylammonio]-1-propanesulfonate) in white, opaque, low-volume 384-well plates. Test compounds were diluted to a 100× final concentration in 100% DMSO. Then, 5 μl of ACE2-Fc/protein A acceptor beads was added first to the plate, followed by 100 nl of test compounds and then 5 μl of CoV-Spike-RBD-HIS/nickel chelate donor beads. Test compounds were added to each well using a Janus Nanohead tool (PerkinElmer, Waltham, MA, USA). For each experiment, all conditions were performed in duplicate. Following 2 h of incubation at room temperature, AlphaScreen fluorescent signals were measured using a ClarioStar plate reader (BMG Labtech, Cary, NC, USA). Data were normalized to percentage inhibition, where 100% equaled the AlphaScreen signal in the absence of SARS-CoV-2-spike-RBD-His and 0% equaled the AlphaScreen signal in the presence of both proteins and DMSO alone.

PD-1 binding to PD-L1 was also determined using AlphaScreen technology. First, 0.5 nM human PD-L1-Fc (Sino Biological) was incubated with 5 nM His-tagged human PD-1 (Sino Biological) in the presence of 5 μg/ml protein A AlphaScreen acceptor beads and 5 μg/ml nickel chelate donor beads in a total volume of 10 μl of 20 mM HEPES (pH 7.4), 150 mM NaCl, and 0.005% Tween in white, opaque, low-volume 384-well plates. Next, 5 μl of PD-L1-Fc/protein A acceptor beads was added first to the plate, followed by 100 nl of test compound prepared as described above, followed by 5 μl of PD-1-His/nickel chelate donor beads. For each experiment, all conditions were performed in duplicate. Following 2 h of incubation at room temperature, data were collected as described above and normalized to percentage inhibition, where 100% equaled the AlphaScreen signal in the absence of PD-1-His, and 0% equaled the AlphaScreen signal in the presence of both proteins and 0.1% DMSO alone.

### Generation of M^pro^ protein.

The codon-optimized gene for SARS-CoV-2 M^pro^ (or 3CL^pro^) (GenBank accession no. QHD43415.1; amino acids [aa] 3264 to 3567) from strain BetaCoV/Wuhan/WIV04/2019 was ordered from IDT (Coralville, IA, USA) and cloned into a HIS-SUMO expression vector (a modified pET-DUET; Novagen, Madison, WI, USA). After transformation into BL21(DE3), the HIS-SUMO-M^pro^ fusion protein was expressed using the autoinduction method (56) with 500-ml cultures at 22°C overnight. Cell pellets were resuspended in a buffer containing 25 mM Tris, pH 8.5, 20 mM imidazole, 200 mM NaCl, and 5 mM β-mercaptoethanol and lysed using sonication and lysozyme and centrifuged at high speed. The supernatant was applied to a Ni-NTA (nickel-nitrilotriacetic acid) column at 4°C and washed with the resuspension buffer. The fusion protein was then eluted using a buffer of 300 mM imidazole, 200 mM NaCl, and 5 mM β-mercaptoethanol, concentrated and applied to a gel filtration column (HiLoad 26/60 Superdex 75; Cytiva, Marlborough, MA) and equilibrated with the resuspension buffer. Fractions with >90% purity were pooled and incubated with SUMO protease at 4°C overnight. After cleavage, the digested protein solution was applied twice to a 5-ml HIS-TRAP Ni-NTA column (Cytiva) to remove the HIS-SUMO and SUMO protease, and the flowthrough was collected. Finally, the protein was concentrated and applied to a second gel filtration column (HiLoad 26/60 Superdex 75; Cytiva) equilibrated with 25 mM HEPES, pH 7.5, 150 mM NaCl, and 2 mM TCEP. Purity (>95%) was confirmed using an SDS-PAGE gel.

### M^pro^ enzymatic assays.

Protease activity of recombinant M^pro^ was measured using the quenched fluorogenic substrate {DABCYL}-Lys-Thr- Ser-Ala-Val-Leu-Gln-Ser-Gly-Phe-Arg-Lys-Met-Glu-(EDANS)-NH2 (Bachem, Vista, CA, USA). Then, 5 μl of 25 nM M^pro^ diluted in assay buffer (25 mM HEPES [pH 7.4]), 150 mM NaCl, 5 mM DTT, and 0.005% Tween) was dispensed into black, low-volume, 384-well plates. Test compounds were serially diluted into 100% DMSO, and 0.1 μl was added to the assay using a Janus MDT Nanohead tool (PerkinElmer). Assays were initiated by addition of 5 μl of 5 μM fluorogenic substrate, and fluorescence at 355 nm excitation and 460 nm emission was monitored every 5 min for 50 min using an Envision plate reader (PerkinElmer). The rate of substrate cleavage was determined using linear regression of the raw data values obtained during the time course. The slopes of these progress curves were then normalized to percentage inhibition, where 100% equaled the rate in the absence of M^pro^ (which was typically 0), and 0% equaled the rate of cleavage in the presence of M^pro^ and 0.1% DMSO.

### Generation of VSVΔG-S-GFP pseudoviruses.

SARS-CoV-2 SWE/01/2020 spike cDNA (containing 100% sequence identity to USA-WA1/2020 in the RBD and 99.9% identity overall) was obtained as a gift from Stephen J. Elledge. B.1.1.7 spike cDNA was generated from SWE/01/2020 spike cDNA by standard PCR mutagenesis, and B.1.351 spike cDNA was synthesized (GenScript, Piscataway, NJ, USA). Pseudoviruses were generated in BHK-21/WI-2 cells using a pseudotyped ΔG-GFP (G*ΔG-GFP) recombinant VSV (rVSV) (Kerafast, Boston, MA, USA) in addition to spike sequences cloned into the paT7-Spike plasmid as described previously (22). Then, 2 h following chemical transfection of spike plasmid, cells were infected with ΔG-GFP (G*ΔG-GFP) rVSV. Following 24 h of incubation, supernatants were harvested, aliquoted, and stored at −80°C.

### Pseudovirus-based infectivity assays.

A total of 12,500 Vero-E6 cells resuspended in 12.5 μl D10+ were plated in 384-μl plates, followed by addition of 6.25 μl of test agents diluted in D10+ at 4× the desired final concentration plus 6.25 μl of undiluted pseudovirus stock (total, 25-μl reaction volumes). All experimental conditions with test agents were performed in duplicate, and control cells with or without pseudovirus in the presence of 0.1% DMSO vehicle control were tested 4-fold. Cells were incubated at 37°C and 5% CO_2_ for 24 h. Cells were then stained with 25 μl of 5 μg/ml Hoechst 33342 (Sigma-Aldrich), incubated for 20 min, and fixed with paraformaldehyde to a 2% final concentration. High-content imaging was then performed across 15 nonoverlapping images per well using a Nikon Eclipse Ti inverted microscope and Nikon NIS Elements AR software v. 5.30.02 (Nikon Americas, Inc., Melville, NY, USA). For each image, cell nuclei and GFP-positive cells were counted, with GFP-positive cells reported as the percentage of total nuclei within each image.

### Resazurin cell viability assay in uninfected cells.

A total of 20,000 Vero-E6 cells were plated in 96-well plates and incubated overnight before addition of compounds at defined concentrations; 0.1% DMSO vehicle control was added to wells in the absence of test compounds. All experimental conditions were performed in duplicate. Cells were then incubated at 37°C and 5% CO_2_ for 4 days before addition of resazurin (Sigma-Aldrich) to a final concentration of 20 μg/ml. Cells were incubated for an additional 4 h before fluorescence intensity was measured using a ClarioStar plate reader (BMG Labtech). Background fluorescence was subtracted from wells containing resazurin and D10+ medium but no cells.

### Generation of SARS-CoV-2 viruses.

First, 3 × 10^6^ Vero-E6 cells were incubated in 15 ml of D10+ medium for 24 h. Cells were then washed and replaced with 10 ml of D10+ containing virus at a multiplicity of infection (MOI) of 0.001. Cells were incubated for 5 to 7 days until clear CPE was observed throughout the flask. Medium was harvested and split into 250-μl aliquots for storage at −80°C.

To determine the titer of virus stocks, Vero-E6 cells were first plated to 20,000 cells per well in a 96-well format in D10+ medium and incubated for 24 h. Following incubation, cells were incubated in fresh D10+ containing 5-fold serial dilutions of a thawed virus aliquot (8 total dilutions, 5-fold replicates) and incubated for an additional 4 days. Wells were then scored for the presence of CPE. TCID_50_s were calculated using the Reed-Muench method.

### Viral CPE scoring-based assays.

Vero-E6 cells were plated in D10+ to 20,000 cells per well in a 96-well format and incubated for 24 h. Following incubation, compounds were added to final concentrations in 8-fold replicates and incubated for an additional 2 h before addition of 50× TCID_50_ of virus. Each 96-well plate further contained uninfected cells and infected cells with 0.1% DMSO vehicle control in 4-fold replicates. Cells were incubated for an additional 4 days, at which point all wells were scored for the presence of CPE by a user blinded to the identity of the wells.

### Viral CPE quantitative assay (i.e., cell viability restoration).

Assays were performed as described for viral CPE scoring-based assays but with addition of 150× TCID_50_ of virus at 2 h following compound addition. Cells were then incubated for an additional 4 to 7 days, at which point cells were treated with resazurin for 4 h, fixed with paraformaldehyde to a final concentration of 4%, and incubated at room temperature for at least 30 min to inactivate virus. Fluorescence intensity was then measured using a ClarioStar plate reader. Background fluorescence was subtracted from wells containing resazurin and D10+ medium but no cells.

### Virus yield reduction assays.

Vero-E6 cells were plated in D10+ to 5,000 cells per well in a 384-well format and incubated for 24 h. Following incubation, supernatants from 4 of 8 replicates from viral CPE scoring based-assay experimental conditions were combined and added to cells at 1:10 final concentration dilutions. Each 384-well plate further contained supernatant dilutions from uninfected cells and infected cells with 0.1% DMSO vehicle control. Cells were incubated for 48 h and then fixed in a final concentration of 4% paraformaldehyde for at least 30 min to inactivate virus. Immunostaining was then performed using primary anti-SARS-CoV-2 nucleocapsid primary antibody (HL344; GeneTex, Irvine, CA, USA) at a 1:1,000 concentration and goat anti-rabbit IgG Alexa Fluor 555 secondary antibody at a 1:2,000 concentration (Thermo Fisher, Waltham, MA, USA). Cells were also counterstained with 1 μg/ml Hoechst. High-content imaging was performed across 9 nonoverlapping images per well using a Nikon Eclipse Ti inverted microscope as described above.

### Data analysis.

For all studies, 50% effective concentrations were calculated using nonlinear regression of a one-side binding model using Prism v. 8.4.3 (GraphPad, San Diego, CA, USA). All data are presented as the mean ± SEM from at least 3 independent experiments, with the exception of data from virus yield reduction assays, which are presented as the mean ± SD from 2 independent experiments. Statistical significance was determined using the ratio paired *t* test function in GraphPad Prism v. 8.4.3, with a *P* value of <0.05 considered significant.

## References

[1] Xiu S, Dick A, Ju H, Mirzaie S, Abdi F, Cocklin S, Zhan P, Liu X. 2020. Inhibitors of SARS-CoV-2 entry: current and future opportunities. J Med Chem 63:12256–12274. 10.1021/acs.jmedchem.0c00502. 32539378 PMC7315836

[2] Hoffmann M, Kleine-Weber H, Schroeder S, Krüger N, Herrler T, Erichsen S, Schiergens TS, Herrler G, Wu NH, Nitsche A, Müller MA, Drosten C, Pöhlmann S. 2020. SARS-CoV-2 cell entry depends on ACE2 and TMPRSS2 and is blocked by a clinically proven protease inhibitor. Cell 181:271–280. 10.1016/j.cell.2020.02.052. 32142651 PMC7102627

[3] Wrapp D, Wang N, Corbett KS, Goldsmith JA, Hsieh CL, Abiona O, Graham BS, McLellan JS. 2020. Cryo-EM structure of the 2019-nCoV spike in the prefusion conformation. Science 367:1260–1263. 10.1126/science.abb2507. 32075877 PMC7164637

[4] Hou YJ, Chiba S, Halfmann P, Ehre C, Kuroda M, Dinnon KH, Leist SR, Schäfer A, Nakajima N, Takahashi K, Lee RE, Mascenik TM, Graham R, Edwards CE, Tse LV, Okuda K, Markmann AJ, Bartelt L, de Silva A, Margolis DM, Boucher RC, Randell SH, Suzuki T, Gralinski LE, Kawaoka Y, Baric RS. 2020. SARS-CoV-2 D614G variant exhibits efficient replication *ex vivo* and transmission *in vivo*. Science 370:1464–1468. 10.1126/science.abe8499. 33184236 PMC7775736

[5] Wang P, Nair MS, Liu L, Iketani S, Luo Y, Guo Y, Wang M, Yu J, Zhang B, Kwong PD, Graham BS, Mascola JR, Chang JY, Yin MT, Sobieszczyk M, Kyratsous CA, Shapiro L, Sheng Z, Huang Y, Ho DD. 2021. Antibody resistance of SARS-CoV-2 variants B.1.351 and B.1.1.7. Nature 593:130–135. 10.1038/s41586-021-03398-2. 33684923

[6] Supasa P, Zhou D, Dejnirattisai W, Liu C, Mentzer AJ, Ginn HM, Zhao Y, Duyvesteyn HME, Nutalai R, Tuekprakhon A, Wang B, Paesen GC, Slon-Campos J, López-Camacho C, Hallis B, Coombes N, Bewley KR, Charlton S, Walter TS, Barnes E, Dunachie SJ, Skelly D, Lumley SF, Baker N, Shaik I, Humphries HE, Godwin K, Gent N, Sienkiewicz A, Dold C, Levin R, Dong T, Pollard AJ, Knight JC, Klenerman P, Crook D, Lambe T, Clutterbuck E, Bibi S, Flaxman A, Bittaye M, Belij-Rammerstorfer S, Gilbert S, Hall DR, Williams MA, Paterson NG, James W, Carroll MW, Fry EE, Mongkolsapaya J, et al. 2021. Reduced neutralization of SARS-CoV-2 B.1.1.7 variant by convalescent and vaccine sera. Cell 184:2201–2211.e7. 10.1016/j.cell.2021.02.033. 33743891 PMC7891044

[7] Li Q, Nie J, Wu J, Zhang L, Ding R, Wang H, Zhang Y, Li T, Liu S, Zhang M, Zhao C, Liu H, Nie L, Qin H, Wang M, Lu Q, Li X, Liu J, Liang H, Shi Y, Shen Y, Xie L, Zhang L, Qu X, Xu W, Huang W, Wang Y. 2021. SARS-CoV-2 501Y.V2 variants lack higher infectivity but do have immune escape. Cell 184:2362–2371.e9. 10.1016/j.cell.2021.02.042. 33735608 PMC7901273

[8] Zhou D, Dejnirattisai W, Supasa P, Liu C, Mentzer AJ, Ginn HM, Zhao Y, Duyvesteyn HME, Tuekprakhon A, Nutalai R, Wang B, Paesen GC, Lopez-Camacho C, Slon-Campos J, Hallis B, Coombes N, Bewley K, Charlton S, Walter TS, Skelly D, Lumley SF, Dold C, Levin R, Dong T, Pollard AJ, Knight JC, Crook D, Lambe T, Clutterbuck E, Bibi S, Flaxman A, Bittaye M, Belij-Rammerstorfer S, Gilbert S, James W, Carroll MW, Klenerman P, Barnes E, Dunachie SJ, Fry EE, Mongkolsapaya J, Ren J, Stuart DI, Screaton GR. 2021. Evidence of escape of SARS-CoV-2 variant B.1.351 from natural and vaccine-induced sera. Cell 184:2348–2361.e6. 10.1016/j.cell.2021.02.037. 33730597 PMC7901269

[9] Islam MT, Sarkar C, El-Kersh DM, Jamaddar S, Uddin SJ, Shilpi JA, Mubarak MS. 2020. Natural products and their derivatives against coronavirus: a review of the non-clinical and pre-clinical data. Phytother Res 34:2471–2492. 10.1002/ptr.6700. 32248575

[10] Vougogiannopoulou K, Corona A, Tramontano E, Alexis MN, Skaltsounis AL. 2021. Natural and nature-derived products targeting human coronaviruses. Molecules 26:448. 10.3390/molecules26020448. 33467029 PMC7831024

[11] Zulfiqar B, Jones AJ, Sykes ML, Shelper TB, Davis RA, Avery VM. 2017. Screening a natural product-based library against kinetoplastid parasites. Molecules 22:1715. 10.3390/molecules22101715. PMC6151456 29023425

[12] Kumar R, Bidgood CL, Levrier C, Gunter JH, Nelson CC, Sadowski MC, Davis RA. 2020. Synthesis of a unique psammaplysin F library and functional evaluation in prostate cancer cells by multiparametric quantitative single cell imaging. J Nat Prod 83:2357–2366. 10.1021/acs.jnatprod.0c00121. 32691595

[13] Yasgar A, Jadhav A, Simeonov A, Coussens NP. 2016. AlphaScreen-based assays: ultra-high-throughput screening for small-molecule inhibitors of challenging enzymes and protein-protein interactions. Methods Mol Biol 1439:77–98. 10.1007/978-1-4939-3673-1_5. 27316989 PMC5444910

[14] Bray MA, Carpenter A. 2017. Advanced assay development guidelines for image-based high content screening and analysis. *In* Markossian S, Sittampalam GS, Grossman A, Brimacombe K, Arkin M, Auld D, Austin CP, Baell J, Caaveiro JMM, Chung TDY, Coussens NP, Dahlin JL, Devanaryan V, Foley TL, Glicksman M, Hall MD, Haas JV, Hoare SRJ, Inglese J, Iversen PW, Kahl SD, Kales SC, Kirshner S, Lal-Nag M, Li Z, McGee J, McManus O, Riss T, Saradjian P, Trask OJ Jr, Weidner JR, Wildey MJ, Xia M, Xu X, (ed), Assay guidance manual. Eli Lilly & Company and the National Center for Advancing Translational Sciences, Bethesda, MD. ncbi.nlm.nih.gov/books/NBK126174/23469374

[15] Starr TN, Greaney AJ, Addetia A, Hannon WW, Choudhary MC, Dingens AS, Li JZ, Bloom JD. 2021. Prospective mapping of viral mutations that escape antibodies used to treat COVID-19. Science 371:850–854. 10.1126/science.abf9302. 33495308 PMC7963219

[16] Goufo P, Singh RK, Cortez I. 2020. A reference list of phenolic compounds (including stilbenes) in grapevine (*Vitis vinifera* L.) roots, woods, canes, stems, and leaves. Antioxidants (Basel) 9:398. 10.3390/antiox9050398. PMC7278806 32397203

[17] Lung J, Hung MS, Lin YC, Hung CH, Chen CC, Lee KD, Tsai YH. 2020. Virtual screening and in vitro evaluation of PD-1 dimer stabilizers for uncoupling PD-L1/PD-L1 interaction from natural products. Molecules 25:5293. 10.3390/molecules25225293. PMC7696397 33202823

[18] Skalniak L, Zak KM, Guzik K, Magiera K, Musielak B, Pachota M, Szelazek B, Kocik J, Grudnik P, Tomala M, Krzanik S, Pyrc K, Dömling A, Dubin G, Holak TA. 2017. Small-molecule inhibitors of PD-1/PD-L1 immune checkpoint alleviate the PD-L1-induced exhaustion of T-cells. Oncotarget 8:72167–72181. 10.18632/oncotarget.20050. 29069777 PMC5641120

[19] Sharma P, Vijayan V, Pant P, Sharma M, Vikram N, Kaur P, Singh TP, Sharma S. 2020. Identification of potential drug candidates to combat COVID-19: a structural study using the main protease (Mpro) of SARS-CoV-2. J Biomol Struct Dyn 39:1–11. 10.1080/07391102.2020.1798286. PMC7441759 32741313

[20] Ma C, Sacco MD, Hurst B, Townsend JA, Hu Y, Szeto T, Zhang X, Tarbet B, Marty MT, Chen Y, Wang J. 2020. Boceprevir, GC-376, and calpain inhibitors II, XII inhibit SARS-CoV-2 viral replication by targeting the viral main protease. Cell Res 30:678–692. 10.1038/s41422-020-0356-z. 32541865 PMC7294525

[21] Fu L, Ye F, Feng Y, Yu F, Wang Q, Wu Y, Zhao C, Sun H, Huang B, Niu P, Song H, Shi Y, Li X, Tan W, Qi J, Gao GF. 2020. Both boceprevir and GC376 efficaciously inhibit SARS-CoV-2 by targeting its main protease. Nat Commun 11:4417. 10.1038/s41467-020-18233-x. 32887884 PMC7474075

[22] Whitt MA. 2010. Generation of VSV pseudotypes using recombinant ΔG-VSV for studies on virus entry, identification of entry inhibitors, and immune responses to vaccines. J Virol Methods 169:365–374. 10.1016/j.jviromet.2010.08.006. 20709108 PMC2956192

[23] Li W, Moore MJ, Vasilieva N, Sui J, Wong SK, Berne MA, Somasundaran M, Sullivan JL, Luzuriaga K, Greenough TC, Choe H, Farzan M. 2003. Angiotensin-converting enzyme 2 is a functional receptor for the SARS coronavirus. Nature 426:450–454. 10.1038/nature02145. 14647384 PMC7095016

[24] Conceicao C, Thakur N, Human S, Kelly JT, Logan L, Bialy D, Bhat S, Stevenson-Leggett P, Zagrajek AK, Hollinghurst P, Varga M, Tsirigoti C, Tully M, Chiu C, Moffat K, Silesian AP, Hammond JA, Maier HJ, Bickerton E, Shelton H, Dietrich I, Graham SC, Bailey D. 2020. The SARS-CoV-2 spike protein has a broad tropism for mammalian ACE2 proteins. PLoS Biol 18:e3001016. 10.1371/journal.pbio.3001016. 33347434 PMC7751883

[25] Ren X, Glende J, Al-Falah M, de Vries V, Schwegmann-Wessels C, Qu X, Tan L, Tschernig T, Deng H, Naim HY, Herrler G. 2006. Analysis of ACE2 in polarized epithelial cells: surface expression and function as receptor for severe acute respiratory syndrome-associated coronavirus. J Gen Virol 87:1691–1695. 10.1099/vir.0.81749-0. 16690935

[26] Case JB, Bailey AL, Kim AS, Chen RE, Diamond MS. 2020. Growth, detection, quantification, and inactivation of SARS-CoV-2. Virology 548:39–48. 10.1016/j.virol.2020.05.015. 32838945 PMC7293183

[27] Gorshkov K, Chen CZ, Bostwick R, Rasmussen L, Tran BN, Cheng Y-S, Xu M, Pradhan M, Henderson M, Zhu W, Oh E, Susumu K, Wolak M, Shamim K, Huang W, Hu X, Shen M, Klumpp-Thomas C, Itkin Z, Shinn P, Carlos de la Torre J, Simeonov A, Michael SG, Hall MD, Lo DC, Zheng W. 2021. The SARS-CoV-2 cytopathic effect is blocked by lysosome alkalizing small molecules. ACS Infect Dis 7:1389–1408. 10.1021/acsinfecdis.0c00349. 33346633 PMC7771250

[28] Pruijssers AJ, George AS, Schäfer A, Leist SR, Gralinksi LE, Dinnon KH, 3rd, Yount BL, Agostini ML, Stevens LJ, Chappell JD, Lu X, Hughes TM, Gully K, Martinez DR, Brown AJ, Graham RL, Perry JK, Du Pont V, Pitts J, Ma B, Babusis D, Murakami E, Feng JY, Bilello JP, Porter DP, Cihlar T, Baric RS, Denison MR, Sheahan TP. 2020. Remdesivir inhibits SARS-CoV-2 in human lung cells and chimeric SARS-CoV expressing the SARS-CoV-2 RNA polymerase in mice. Cell Rep 32:107940. 10.1016/j.celrep.2020.107940. 32668216 PMC7340027

[29] Vuong W, Khan MB, Fischer C, Arutyunova E, Lamer T, Shields J, Saffran HA, McKay RT, van Belkum MJ, Joyce MA, Young HS, Tyrrell DL, Vederas JC, Lemieux MJ. 2020. Feline coronavirus drug inhibits the main protease of SARS-CoV-2 and blocks virus replication. Nat Commun 11:4282. 10.1038/s41467-020-18096-2. 32855413 PMC7453019

[30] Painter GR, Natchus MG, Cohen O, Holman W, Painter WP. 2021. Developing a direct acting, orally available antiviral agent in a pandemic: the evolution of molnupiravir as a potential treatment for COVID-19. Curr Opin Virol 50:17–22. 10.1016/j.coviro.2021.06.003. 34271264 PMC8277160

[31] Boras B, Jones RM, Anson BJ, Arenson D, Aschenbrenner L, Bakowski MA, Beutler N, Binder J, Chen E, Eng H, Hammond H, Hammond J, Haupt RE, Hoffman R, Kadar EP, Kania R, Kimoto E, Kirkpatrick MG, Lanyon L, Lendy EK, Lillis JR, Logue J, Luthra SA, Ma C, Mason SW, McGrath ME, Noell S, Obach RS, O’Brien MN, O’Connor R, Ogilvie K, Owen D, Pettersson M, Reese MR, Rogers TF, Rossulek MI, Sathish JG, Shirai N, Steppan C, Ticehurst M, Updyke LW, Weston S, Zhu Y, Wang J, Chatterjee AK, Mesecar AD, Frieman MB, Anderson AS, Allerton C. 2021. Discovery of a novel inhibitor of coronavirus 3CL protease for the potential treatment of COVID-19. bioRxiv 10.1101/2020.09.12.293498. PMC8523698 34663813

[32] Tanaka T, Ito T, Ido Y, Son TK, Nakaya K, Iinuma M, Ohyama M, Chelladurai V. 2000. Stilbenoids in the stem bark of *Hopea parviflora*. Phytochemistry 53:1015–1019. 10.1016/s0031-9422(00)00019-4. 10820823

[33] Davis RA, Beattie KD, Xu M, Yang X, Yin S, Holla H, Healy PC, Sykes M, Shelper T, Avery VM, Elofsson M, Sundin C, Quinn RJ. 2014. Solving the supply of resveratrol tetramers from Papua New Guinean rainforest *Anisoptera* species that inhibit bacterial type III secretion systems. J Nat Prod 77:2633–2640. 10.1021/np500433z. 25405587

[34] Lin YS, Chen CR, Wu WH, Wen CL, Chang CI, Hou WC. 2015. Anti-α-glucosidase and anti-dipeptidyl peptidase IV activities of extracts and purified compounds from *Vitis thunbergii* var. *taiwaniana*. J Agric Food Chem 63:6393–6401. 10.1021/acs.jafc.5b02069. 26138774

[35] Moriyama H, Moriyama M, Ninomiya K, Morikawa T, Hayakawa T. 2016. Inhibitory effects of oligostilbenoids from the bark of *Shorea roxburghii* on malignant melanoma cell growth: implications for novel topical anticancer candidates. Biol Pharm Bull 39:1675–1682. 10.1248/bpb.b16-00420. 27725445

[36] Chang CI, Chien WC, Huang KX, Hsu JL. 2017. Anti-inflammatory effects of vitisinol A and four other oligostilbenes from *Ampelopsis brevipedunculata* var. *Hancei*. Molecules 22:1195. 10.3390/molecules22071195. PMC6152071 28714918

[37] Ninomiya K, Chaipech S, Kunikata Y, Yagi R, Pongpiriyadacha Y, Muraoka O, Morikawa T. 2017. Quantitative determination of stilbenoids and dihydroisocoumarins in *Shorea roxburghii* and evaluation of their hepatoprotective activity. Int J Mol Sci 18:451. 10.3390/ijms18020451. PMC5343985 28230758

[38] Schrader KK, Ibrahim MA, Abd-Alla HI, Cantrell CL, Pasco DS. 2018. Antibacterial activities of metabolites from *Vitis rotundifolia* (Muscadine) roots against fish pathogenic bacteria. Molecules 23:2761. 10.3390/molecules23112761. PMC6278413 30366372

[39] Prabha B, Sini S, Priyadarshini TS, Sasikumar P, Gopalan G, Joseph JP, Jithin MM, Sivan VV, Jayamurthy P, Radhakrishnan KV. 2021. Anti-inflammatory effect and mechanism of action of ellagic acid-3,3′,4-trimethoxy-4′-*O*-α-L-rhamnopyranoside isolated from *Hopea parviflora* in lipopolysaccharide-stimulated RAW 264.7 macrophages. Nat Prod Res 35:3156–3160. 10.1080/14786419.2019.1690486. 31711318

[40] Aja I, Ruiz-Larrea MB, Courtois A, Krisa S, Richard T, Ruiz-Sanz JI. 2020. Screening of natural stilbene oligomers from *Vitis vinifera* for anticancer activity on human hepatocellular carcinoma cells. Antioxidants (Basel) 9:469. 10.3390/antiox9060469. PMC7346113 32492881

[41] Ohyama M, Tanaka T, Ito T, Iinuma M, Bastow KF, Lee KH. 1999. Antitumor agents 200. Cytotoxicity of naturally occurring resveratrol oligomers and their acetate derivatives. Bioorg Med Chem Lett 9:3057–3060. 10.1016/s0960-894x(99)00520-x. 10571175

[42] Zetterström CE, Hasselgren J, Salin O, Davis RA, Quinn RJ, Sundin C, Elofsson M. 2013. The resveratrol tetramer (-)-hopeaphenol inhibits type III secretion in the gram-negative pathogens *Yersinia pseudotuberculosis* and *Pseudomonas aeruginosa*. PLoS One 8:e81969. 10.1371/journal.pone.0081969. 24324737 PMC3853165

[43] Schnee S, Queiroz EF, Voinesco F, Marcourt L, Dubuis PH, Wolfender JL, Gindro K. 2013. *Vitis vinifera* canes, a new source of antifungal compounds against *Plasmopara viticola*, *Erysiphe necator*, and *Botrytis cinerea*. J Agric Food Chem 61:5459–5467. 10.1021/jf4010252. 23730921

[44] Ito T, Hayashi K, Nishiguchi M, Hayashi T, Iinuma M. 2018. Resveratrol oligomer C-glucosides and anti-viral resveratrol tetramers isolated from the stem bark of *Shorea uliginosa*. Phytochem Lett 28:1–7. 10.1016/j.phytol.2018.07.026.

[45] Mattio LM, Catinella G, Pinto A, Dallavalle S. 2020. Natural and nature-inspired stilbenoids as antiviral agents. Eur J Med Chem 202:112541. 10.1016/j.ejmech.2020.112541. 32652408 PMC7335248

[46] Tabata Y, Takano K, Ito T, Iinuma M, Yoshimoto T, Miura H, Kitao Y, Ogawa S, Hori O. 2007. Vaticanol B, a resveratrol tetramer, regulates endoplasmic reticulum stress and inflammation. Am J Physiol Cell Physiol 293:C411–418. 10.1152/ajpcell.00095.2007. 17475668

[47] Morikawa T, Chaipech S, Matsuda H, Hamao M, Umeda Y, Sato H, Tamura H, Ninomiya K, Yoshikawa M, Pongpiriyadacha Y, Hayakawa T, Muraoka O. 2012. Anti-hyperlipidemic constituents from the bark of *Shorea roxburghii*. J Nat Med 66:516–524. 10.1007/s11418-011-0619-6. 22261856

[48] Morikawa T, Chaipech S, Matsuda H, Hamao M, Umeda Y, Sato H, Tamura H, Kon’i H, Ninomiya K, Yoshikawa M, Pongpiriyadacha Y, Hayakawa T, Muraoka O. 2012. Antidiabetogenic oligostilbenoids and 3-ethyl-4-phenyl-3,4-dihydroisocoumarins from the bark of *Shorea roxburghii*. Bioorg Med Chem 20:832–840. 10.1016/j.bmc.2011.11.067. 22209731

[49] Wahedi HM, Ahmad S, Abbasi SW. 2021. Stilbene-based natural compounds as promising drug candidates against COVID-19. J Biomol Struct Dyn 39:3225–3234. 10.1080/07391102.2020.1762743. 32345140

[50] Gangadevi S, Badavath VN, Thakur A, Yin N, De Jonghe S, Acevedo O, Jochmans D, Leyssen P, Wang K, Neyts J, Yujie T, Blum G. 2021. Kobophenol A inhibits binding of host ACE2 receptor with spike RBD domain of SARS-CoV-2, a lead compound for blocking COVID-19. J Phys Chem Lett 12:1793–1802. 10.1021/acs.jpclett.0c03119. 33577324

[51] Pasquereau S, Nehme Z, Ahmad SH, Daouad F, Van Assche JV, Wallet C, Schwartz C, Rohr O, Morot-Bizot S, Herbein G. 2021. Resveratrol inhibits HCoV-229E and SARS-CoV-2 coronavirus replication *in vitro*. Viruses 13:354. 10.3390/v13020354. 33672333 PMC7926471

[52] Baell JB. 2016. Feeling nature’s PAINS: natural products, natural product drugs, and pan assay interference compounds (PAINS). J Nat Prod 79:616–628. 10.1021/acs.jnatprod.5b00947. 26900761

[53] Lü JM, Lin PH, Yao Q, Chen C. 2010. Chemical and molecular mechanisms of antioxidants: experimental approaches and model systems. J Cell Mol Med 14:840–860. 10.1111/j.1582-4934.2009.00897.x. 19754673 PMC2927345

[54] D’Archivio M, Filesi C, Varì R, Scazzocchio B, Masella R. 2010. Bioavailability of the polyphenols: status and controversies. Int J Mol Sci 11:1321–1342. 10.3390/ijms11041321. 20480022 PMC2871118

[55] Marín L, Miguélez EM, Villar CJ, Lombó F. 2015. Bioavailability of dietary polyphenols and gut microbiota metabolism: antimicrobial properties. Biomed Res Int 2015:905215. 10.1155/2015/905215. 25802870 PMC4352739

[56] Studier FW. 2005. Protein production by auto-induction in high density shaking cultures. Protein Expr Purif 41:207–234. 10.1016/j.pep.2005.01.016. 15915565

