# Supplementary material for: The Natural Stilbenoid (–)-Hopeaphenol Inhibits Cellular Entry of SARS-CoV-2 USA-WA1/2020, B.1.1.7, and B.1.351 Variants
Source: Antimicrob Agents Chemother. 2021 Nov 17;65(12):e00772-21. doi: 10.1128/AAC.00772-21 (PMC8597786; doi:10.1128/AAC.00772-21)
Supplement: Supplemental file 1 — Supplemental material. Download aac.00772-21-s0001.pdf, PDF file, 0.1 MB [file aac.00772-21-s0001.pdf]

Supplemental Table 1. Summary of all high-content imaging data obtained from pseudovirus transduction assays.

Spike sequence	Compound (50 µM)	Replicate	N Nuclei	N GFP+ nuclei	% GFP+ nuclei	Average	SEM	p value vs. infected, no-drug
Uninfected	No drug	1	11362	1	0.01%	0.01%	0.00%	
		2	13479	1	0.01%			
		3	12014	2	0.01%			
USA-WA1/2020	No drug	1	10155	266	2.63%	2.45%	0.17%	
		2	11005	287	2.61%			
		3	11311	239	2.11%			
	Hopeaphenol	1	16077	120	0.75%	0.69%	0.03%	**3.5 x 10-3**
		2	14310	93	0.65%			
		3	11206	76	0.68%			
	Vatalbinoside A	1	12729	154	1.21%	0.99%	0.41%	0.16
		2	11733	183	1.56%			
		3	5774	12	0.21%			
	Vaticanol B	1	11652	253	2.17%	2.05%	0.12%	0.26
		2	13822	251	1.82%			
		3	11072	240	2.17%			
	Resveratrol	1	7611	142	1.87%	2.36%	0.36%	0.74
		2	6485	198	3.05%			
		3	5396	116	2.15%			
B.1.1.7	No drug	1	11049	529	4.80%	5.36%	1.59%	
		2	10893	1229	11.62%			
		3	17605	709	4.02%			
		4	12996	396	3.05%			
		5	10218	338	3.32%			
	Hopeaphenol	1	13236	163	1.23%	1.83%	0.73%	**4.0 x 10^{-4}**
		2	14298	575	4.02%			
		3	10285	110	1.07%			
		5	12903	128	0.99%			
	Vatalbinoside A	3	17919	443	2.48%	2.35%	0.18%	0.07
		4	11426	294	2.57%			
		5	10585	211	1.99%			
	Vaticanol B	3	17666	431	2.44%	2.02%	0.22%	**0.002**
		4	12375	211	1.71%			
		5	10256	194	1.89%			
B.1.351	No drug	1	10869	607	5.61%	4.55%	0.64%	
		2	13019	847	6.48%			
		3	19538	710	3.62%			
		4	13665	416	3.04%			
		5	10083	403	3.99%			
	Hopeaphenol	1	13631	99	0.73%	0.93%	0.11%	**0.012**
		2	12749	102	0.80%			
		3	11497	112	0.97%			
		4	12391	153	1.23%			
	Vatalbinoside A	3	18003	293	1.63%	1.87%	0.12%	**0.029**
		4	13408	270	1.99%			
		5	10675	213	1.99%			
	Vaticanol B	3	19456	566	2.93%	3.29%	0.33%	0.35
		4	11705	350	2.99%			
		5	10869	429	3.95%			

Supplemental Table 2. Summary of resazurin-based viability measurements obtained from cells infected with SARS-CoV-2 variants. A.U. = arbitrary units.

Virus	Replicate	Uninfected cell viability (A.U.)		Infected cell viability (A.U.)		% viability vs. uninfected cells	p value
		Average	SD	Average	SD		
WA1/2020	1	6866540	547083	1150510	213694	16.8%	**$< 10^{-4}$**
	2	6475746	532339	1120360	2569071	17.3%	
	3	6943744	836644	1192919	87291	17.2%	
B.1.1.7	1	3130441	335566	2709147	302454	86.5%	**0.007**
	2	3329776	408199	2889745	67793	86.8%	
	3	5272236	129469	4383816	387612	83.1%	
B.1.351	1	3219953	312980	2566185	210668	79.7%	**0.026**
	2	3340315	232577	2601518	139652	77.9%	
	3	4877282	430514	4245177	444644	87.0%	

Supplemental Table 3. Summary of all high-content imaging data obtained from virus yield reduction assays.

Virus	Agent	Concentration	Replicate	N Nuclei	N infected	% infected	Average	SD
Uninfected	No drug		1	3388.5	1	0.0%	0.0%	0.0%
			2	2816.5	0	0.0%		
USA-WA1/2020	No drug		1	2901.75	790.5	27.2%	29.0%	2.5%
			2	2216	683	30.8%		
	REGN10933	0.7 nM	1	3482	0	0.0%	0.0%	0.0%
			2	3583	2	0.1%		
		0.07 nM	1	3596	557	15.5%	15.6%	0.1%
			2	3414	536	15.7%		
		0.007 nM	1	3192	959	30.0%	24.4%	7.9%
			2	3560	671	18.8%		
		0.0007 nM	1	3462	775	22.4%	24.1%	2.4%
			2	3675	946	25.7%		
	Hopeaphenol	50 µM	1	2023	113	5.6%	3.6%	2.8%
			2	1948	33	1.7%		
		30 µM	1	2487	190	7.6%	11.5%	5.4%
			2	2585	396	15.3%		
		10 µM	1	2156	367	17.0%	18.7%	2.4%
			2	2160	441	20.4%		
		3 µM	1	1765	471	26.7%	23.0%	5.2%
			2	2258	438	19.4%		
		1 µM	1	2065	602	29.2%	29.2%	0.0%
			2	1619	472	29.2%		
	Vatalbinoside A	50 µM	1	2257	197	8.7%	11.2%	3.5%
			2	1900	260	13.7%		
		30 µM	1	2626	277	10.5%	9.9%	0.9%
			2	2529	236	9.3%		
		10 µM	1	2765	567	20.5%	16.7%	5.3%
			2	2208	286	13.0%		
		3 µM	1	3057	564	18.4%	17.8%	1.0%
			2	2564	438	17.1%		
		1 µM	1	3381	710	21.0%	17.1%	5.5%
			2	2174	288	13.2%		
	Vaticanol B	50 µM	1	2261	386	17.1%	16.9%	0.3%
			2	2259	376	16.6%		
		30 µM	1	2203	537	24.4%	26.8%	3.5%
			2	1719	504	29.3%		
		10 µM	1	1774	3130	38.6%	30.0%	12.2%
			2	685	670	21.4%		
	Remdesivir	10 µM	1	2431	0	0.0%	0.0%	0.0%
			2	2517	0	0.0%		
		3 µM	1	2799	155	5.5%	8.5%	4.2%
			2	2216	254	11.5%		
		1 µM	1	3186	356	11.2%	13.5%	3.3%
			2	3333	529	15.9%		
		0.3 µM	1	3114	537	17.2%	21.0%	5.3%
			2	2959	732	24.7%		

Virus	Agent	Concentration	Replicate	N Nuclei	N infected	% infected	Average	SD
B.1.1.7	No drug		1	3042.75	414.5	13.6%	12.8%	1.1%
			2	3216.75	387	12.0%		
	REGN10933	0.7 nM	1	4060	0	0.0%	0.0%	0.0%
			2	4394	2	0.0%		
		0.07 nM	1	3760	3	0.1%	0.1%	0.0%
			2	4264	2	0.0%		
		0.007 nM	1	3674	327	8.9%	10.0%	1.5%
			2	4157	458	11.0%		
		0.0007 nM	1	3638	343	9.4%	13.1%	5.2%
			2	2422	407	16.8%		
	Hopeaphenol	50 µM	1	3555	0	0.0%	0.0%	0.0%
			2	3901	1	0.0%		
		20 µM	1	3385	238	7.0%	6.7%	0.5%
			2	4004	255	6.4%		
		10 µM	1	3340	372	11.1%	10.4%	1.1%
			2	3825	367	9.6%		
		3 µM	1	3425	349	10.2%	12.1%	2.7%
			2	3735	521	13.9%		
B.1.351	No drug		1	3215.5	566.5	17.6%	18.1%	0.7%
			2	3229.75	603.25	18.7%		
	REGN10933	7 nM	1	3889	16	0.4%	0.3%	0.2%
			2	4447	6	0.1%		
		0.7 nM	1	3443	497	14.4%	16.6%	3.0%
			2	3712	693	18.7%		
		0.07 nM	1	3599	470	13.1%	14.5%	2.1%
			2	3509	560	16.0%		
		0.007 nM	1	3679	588	16.0%	15.0%	1.4%
			2	2991	417	13.9%		
		0.0007 nM	1	3126	454	14.5%	17.5%	4.2%
			2	2338	480	20.5%		
	Hopeaphenol	50 µM	1	3007	4	0.1%	0.1%	0.1%
			2	3263	2	0.1%		
		20 µM	1	3074	309	10.1%	9.9%	0.2%
			2	3216	314	9.8%		
		10 µM	1	3395	348	10.3%	11.6%	1.9%
			2	3388	437	12.9%		
		3 µM	1	3347	634	18.9%	20.6%	2.4%
			2	3777	843	22.3%		
